# Effects of consumer surface sterilization on diet DNA metabarcoding data of terrestrial invertebrates in natural environments and feeding trials

**DOI:** 10.1002/ece3.7968

**Published:** 2021-08-09

**Authors:** Ana Miller‐ter Kuile, Austen Apigo, Hillary S. Young

**Affiliations:** ^1^ Department of Ecology, Evolution, and Marine Biology University of California Santa Barbara Santa Barbara CA USA

**Keywords:** consumptive interactions, contamination, diet analysis, food web, invertebrates, predator–prey interactions

## Abstract

1. DNA metabarcoding is an emerging tool used to quantify diet in environments and consumer groups where traditional approaches are unviable, including small‐bodied invertebrate taxa. However, metabarcoding of small taxa often requires DNA extraction from full body parts (without dissection), and it is unclear whether surface contamination from body parts alters presumed diet presence or diversity.
2. We examined four different measures of diet (presence, rarefied read abundance, richness, and species composition) for a terrestrial invertebrate consumer (the spider *Heteropoda venatoria*) both collected in its natural environment and fed an offered diet item in contained feeding trials using DNA metabarcoding of full body parts (opisthosomas). We compared diet from consumer individuals surface sterilized to remove contaminants in 10% commercial bleach solution followed by deionized water with a set of unsterilized individuals.
3. We found that surface sterilization did not significantly alter any measure of diet for consumers in either a natural environment or feeding trials. The best‐fitting model predicting diet detection in feeding trial consumers included surface sterilization, but this term was not statistically significant (*β* = −2.3, *p*‐value = .07).
4. Our results suggest that surface contamination does not seem to be a significant concern in this DNA diet metabarcoding study for consumers in either a natural terrestrial environment or feeding trials. As the field of diet DNA metabarcoding continues to progress into new environmental contexts with various molecular approaches, we suggest ongoing context‐specific consideration of the possibility of surface contamination.

## Graphical abstract

In this study, we present results of a study examining the effects of a surface sterilization treatment of invertebrate consumers prior to diet DNA metabarcoding on various metrics of diet. We find that no measures of diet (detection, abundance, richness, or composition) are altered by surface sterilization treatments, suggesting a validation of the use of DNA metabarcoding of small‐bodied consumers to identify diet interactions.

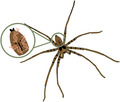

## 1 INTRODUCTION

Biological communities and ecosystem function are shaped by interactions between organisms (Hooper et al., 2005). Among the many interaction types, consumptive interactions (including herbivory, predation, and parasitism) can shape the stability of biologically diverse communities (Delmas et al., 2019). Until recently, consumptive interactions were most often measured by visual observations of feeding or by gut dissection or inspection of fecal contents (Baker et al., 2014; Nielsen et al., 2018), which made it challenging or impossible to conduct diet analyses for many consumer groups. Specifically, these diet analyses are not possible for consumers that (a) are too small for dissection and food identification and (b) have feeding habits or food items which make diet visually unidentifiable (Sheppard & Harwood, 2005). This group of consumers, including terrestrial insects, spiders, and other arthropods, form the base of most terrestrial food webs and are integral to maintaining biodiversity and ecosystem functioning in ecosystems worldwide (Wilson, 1987). For these consumer groups, the use of high‐throughput sequencing is one of the most promising emerging approaches for determining gut contents. High‐throughput sequencing (hereafter referred to as “diet DNA metabarcoding”) can identify a suite of diet species at once and provides a comprehensive and efficient method for determining intrapopulation, intraspecific, and interspecific diets (Lucas et al., 2018; Pompanon et al., 2012; Quéméré et al., 2013; Soininen et al., 2015). These methods have already illuminated new interactions and ecological trends in a variety of environments (e.g., host–parasitoid: (Wirta et al., 2014); plant–herbivore: (Kartzinel et al., 2015); host–parasite: (Schnell et al., 2012); and predator–prey: (Toju & Baba, 2018).

As diet DNA metabarcoding methods continue to advance, however, they need to be validated so that the ecological inference made from them is robust. Focusing on the challenges of small organisms where small body size has limited other diet analysis methods, DNA diet analyses are often performed on full organisms or body parts without gut dissection (e.g., Jacobsen et al., 2018; Toju & Baba, 2018). The necessity to use full organisms or body parts increases the possibility of surface contamination, altering detection and species composition of presumed diet items. Surface sterilization, the use of chemical treatments or physical action to remove surface contaminants, is systematically used in other fields to reduce the risk of contamination in DNA metabarcoding datasets (Burgdorf et al., 2014; Zimmerman & Vitousek, 2012). However, surface sterilization has not been systematically used in diet metabarcoding studies. While some fields have developed informed protocols based on decades of research into best practices and study‐specific considerations (Brown et al., 2018), the field of diet DNA metabarcoding has not developed a similarly systematic approach (e.g., ethanol: Doña et al., 2019, bleach: Anslan et al., 2016, and no sterilization: Jacobsen et al., 2018; Wirta et al., 2014). The lack of systematic surface sterilization in diet DNA metabarcoding when using full individuals or body parts may be due to the desire to avoid DNA destruction in relatively permeable animal cells (Greenstone et al., 2012). However, without considering surface sterilization as a treatment for surface contamination, we have limited ability to confidently assign DNA sequences to ingested diet items (Greenstone et al., 2011, 2012; Linville & Wells, 2002).

In this study, we look at the effects of surface sterilization to remove surface contaminants on our understanding of consumer diets where the DNA of full body parts (no internal dissection) is used for diet DNA metabarcoding. Targeting the CO1 gene region, we produced high‐throughput sequencing results from the full body parts (opisthosomas without gut dissection) of an invertebrate consumer species (the spider, *Heteropoda venatoria*). We surface sterilized half of the consumers prior to DNA extraction using a series of washes in a 1:10 dilution of bleach (10% commercial bleach) and deionized water; we left the other half of consumers unsterilized. We first determined how surface sterilization to remove contaminants impacts presumed diet from consumers collected in their natural environment, comparing surface sterilized individuals to those which were not surface sterilized, to ask whether surface sterilization influences (a) detection, (b) rarefied abundance, (c) richness, and (d) composition of potential diet items. We then performed a laboratory feeding trial, comparing surface sterilized individuals to those which were not surface sterilized to ask whether surface sterilization influenced (a) detection or (b) rarefied abundance of offered diet items. Exploring these questions in natural and contained settings addresses whether surface contamination alters interpretations of feeding interactions and thus whether it should be incorporated into standard protocols in diet metabarcoding.

## 2 MATERIALS AND METHODS

### 2.1 Field site and collections

We conducted fieldwork on Palmyra Atoll National Wildlife Refuge, Northern Line Islands, USA (5°53′N, 162°05′W). Palmyra Atoll has a well‐characterized species list and is relatively species poor, allowing for relatively complete characterization of consumer and diet items (Handler et al., 2007). We targeted a generalist, active hunting spider species (*Heteropoda venatoria*) because (a) it occurs in high abundance on the atoll and is easy to collect, (b) it is a generalist species that feeds on a wide suite of organisms (including spiders, other invertebrates, and two geckos in the genus *Lepidodactylus*), and (c) it is the only species in its family on the atoll, meaning consumer DNA can be differentiated from potential diet DNA. All individuals were stored individually in sterilized containers (Greenstone et al., 2011).

### 2.2 Natural environment consumer collection

In 2015, we collected consumers (*n* = 47) from natural environments, which had fed on available diet items and come into contact with environmental surfaces, to test whether DNA metabarcoding detects diet DNA effectively. Consumers were collected at night via eye shine while they were actively hunting. We collected the first individuals we observed in each survey period and so they represent the distribution of body size and population demographics of this species that actively hunt in that environment. We froze all individuals at −80℃ immediately following collection until surface sterilization and DNA extraction in 2019.

### 2.3 Feeding trial consumer setup and feeding

In 2017, we conducted laboratory trials (*n* = 26) to test whether DNA metabarcoding detects DNA from diet items offered in a contained environment. We created feeding environments from one‐liter plastic yogurt containers with holes for air transfer and placed one *H. venatoria* in each container. After 12 hr, we placed one large grasshopper (*Oxya japonica*, a likely diet item (Handler et al., 2007)) in each container and left all containers for 24 hr. We then froze (−20℃) each *H. venatoria* that had killed the grasshopper (*n* = 25, consumption was not easily detectable and thus not considered in analyses). We cleaned all containers between trials with 10% bleach solution.

To test surface sterilization's efficacy at removing possible contaminants, we used a surface sterilization treatment (Burgdorf et al., 2014; Schulz et al., 1993) on ~half the consumers for each set: those collected from the natural environment and those subjected to controlled feeding trials. We submerged and stirred each (whole) consumer in 10% commercial bleach by volume for 2 min and washed each in deionized water for 2 min. Similar bleach submersion leads to undetectable DNA degradation in similar soft‐exoskeleton consumers (Greenstone et al., 2012; Linville & Wells, 2002). Natural environment consumers (2015) had been frozen at −80°C since collection; we surface sterilized these consumers in a sterilized laminar flow hood in 2019 just before DNA extraction (*n* = 22 surface sterilized, *n* = 25 not surface sterilized; Table 1). We surface sterilized feeding trial consumers (2017) in the laboratory on the atoll in 2017 following freezing at −20℃ and then stored each in individual vials of 95% ethanol in a −20°C freezer until DNA extraction (no −80°C freezer was available at the field station that year) (*n* = 10 surface sterilized; *n* = 14 not surface sterilized). Prior to DNA extraction, we dried all samples for 1–3 hr in a sterilized laminar flow hood and then removed the full opisthosoma (containing the hind gut region) using a sterilized scalpel. Between all steps, tools were sterilized with either ethanol and flame (scalpels and forceps) or 10% bleach (surfaces) between handling each individual.

**TABLE 1:** Sample sizes for successfully extracted and PCR‐amplified samples of surface sterilized and unsterilized *Heteropoda venatoria* individuals in the natural environment and feeding trial studies

Environment	Surface sterilized		Unsterilized	
	Extracted	Amplified and sequenced	Extracted	Amplified and sequenced
Natural environment	22	**18**	25	**19**
Feeding trial	10	**8**	14	**11**

Note

Bold numbers indicate final sample sizes for statistical analyses.

### 2.4 DNA extraction and removal of consumer DNA with AMPure XP beads

We extracted DNA from each consumer following a modified CTAB extraction protocol (Fulton et al., 1995). We quantified DNA using a Qubit (Invitrogen) fluorometer with the high sensitivity double‐stranded DNA quantification kit. We followed Krehenwinkel et al. (2017) to isolate a proportion of lower molecular weight DNA with AMPure XP beads prior to PCR (Appendix S5, Figure S1). We diluted each DNA sample to 20ng/μl (creating a total sample volume of 40μl), mixed each sample using AMPure XP beads (0.75x bead‐to‐DNA ratio), and kept the supernatant. With the supernatant, we precipitated the DNA pellets with isopropanol and 5 M potassium acetate and washed DNA pellets with ethanol (Appendix S6). We quantified this cleaned DNA again using a Qubit fluorometer and diluted all samples to 10 ng/μl prior to PCR steps. All DNA pellets were stored in and diluted with TE buffer.

### 2.5 PCR amplification, library preparation, and sequencing

We amplified the CO1 gene with general metazoan primers (Krehenwinkel et al., 2017; Leray et al., 2013; Yu et al., 2012; Table 2). We performed all PCR preparation steps in a UV‐sterilized biosafety cabinet. We used PCR volumes of 25μl (9μl nuclease free water, 12.5μl GoTaq Green Master Mix (Promega Corp.), 1.25 μl of each of the primers (at 10 mM), and 1 μl of DNA template (at 10 ng/μl)). We ran each sample in duplicate along with duplicated negative samples each PCR run. PCRs are as follows: initial denaturation step at 95℃ for 3 min and then 35 cycles of (a) 95℃ for 30 s, (b) 46℃ for 30 s, and (c) 72℃ for 1 min, followed by a final 5 min at 72℃. We cleaned PCR products with AMPure XP beads at a 0.8x bead‐to‐DNA ratio and resuspended from beads using a 10 mM TRIS buffer.

**TABLE 2:** Primers with Illumina overhang adapters (in bold) used to amplify the CO1 region in this study

Primer	Sequence (5′–3′)	Source
mICOIintF	**TCGTCGGCAGCGTCAGATGTGTATAAGAGACAG**GGWACWGGWTGAACWGTWTAYCCYCC	Yu et al. (2012)
Fol‐degen‐rev	**GTCTCGTGGGCTCGGAGATGTGTATAAGAGACAG**TANACYTCNGGRTGNCCRAARAAYCA	Leray et al. (2013)

We attached Illumina index primers with an additional PCR step following standard protocols (Nextera XT Index Kit v2, Illumina, 2019). We combined duplicate samples for which both duplicates successfully amplified and diluted to a concentration of 5 nM. We multiplexed all samples with one negative control and two fungal clone positive controls (GenBank accession numbers: MG840195 and MG840196; Apigo & Oono, 2018; Clark et al., 2016; Toju et al., 2012). We submitted multiplexed samples for sequencing at the University of California, Santa Barbara Biological Nanostructures Laboratory Genetics Core. Samples were run on an Illumina MiSeq platform (v2 chemistry, 500 cycles, paired‐end reads) with a 15% spike‐in of PhiX. Following sequencing, samples were demultiplexed using Illumina's bcl2fastq conversion software (v2.20) at the Core facility. Our full protocol from DNA extraction through submission for Illumina sequencing can be found in Appendix S6.

### 2.6 Sequence merging, filtering, and clustering with UNOISE3

We merged, filtered (max ee = 1.0), and denoised (clustered) our sequences around amplicon sequence variants (ASVs) using the UNOISE3 algorithm (unoise3 command in the open‐source USEARCH 32‐bit version 11.0.667; Edgar, 2016, Appendix S5, Figure S3). Prior to denoising with UNOISE3, we used cutadapt (version 1.18, Martin, 2011) to remove primers from each sequence. We also repeated analyses with the DADA2 algorithm run through R (dada2 package version 1.1.14.0; Callahan et al., 2016) and with a data cleaning step run through BBSplit (Bushnell, 2019) to remove consumer DNA prior to ASV assignment (because ASV assignment is abundance‐sensitive). We considered analyses from the UNOISE3 algorithm only because UNOISE3 assigned more sequence reads to positive controls than DADA2 (on average, 3× as many reads per positive control) and the cleaning step paired with either DADA2 or UNOISE3 did not increase potential diet DNA detection (summary and comparisons in Appendices S1 and S2).

We created a list of unique ASVs and a matrix of ASV abundances across samples. We matched ASVs to taxonomies in the GenBank and BOLD databases. For GenBank, we used BLAST (version 2.7.1) with the blastn command for taxonomic assignment of each ASV using the computing cluster at UC Santa Barbara, comparing against the GenBank nucleotide database with an evalue of 0.01 (downloaded on 20 November 2019). We visualized and exported taxonomic alignment using MEGAN Community Edition (version 6.18.0, Huson et al., 2016), using default settings (LCA = naïve, MinScore = 50.0, MaxExpected = 0.01, TopPercent = 10.0, MinSupportPercent = 0.05) and selecting the subtree with all possible diet items for this species (Kingdom: Animalia, Clade: Bilateria). For taxonomies which were not assigned below the order level (*n* = 24), we submitted each ASV individually to the BLAST Basic Local Alignment Search Tool and assigned them a family based on the best sequence match in the database, given that the top ten database matches were from the same family. For BOLD taxonomic assignment, we used the BOLD IDEngine of the CO1 gene with Species Level Barcode Records (accessed 5–16 February 2020; 3,825,490 Sequences, 216,704 Species, and 95,537 Interim Species in database) to match each ASV list to taxonomies. We combined taxonomic assignments from both programs and discarded taxonomic assignments that were mismatched at the family level or higher (Elbrecht et al., 2017).

### 2.7 Detection of potential diet items

For consumers from both natural environment and feeding trials, we asked whether surface sterilization altered detection of potential diet items for each consumer. For natural environment consumers, we examined all potential diet items (which could represent either diet or surface contaminants). For feeding trial consumers, we focused our detection analysis on the offered diet item we provided the consumers in the feeding trial environment (*O. japonica*, which all consumers were observed to have killed, but not necessarily ingested). We rarefied (McKnight et al., 2019, Appendix S5, Figure S4) based on the sample with the lowest sequencing depth which had been sequenced with 95%+ sampling completeness based on iNEXT (version 2.0.20) interpolation and extrapolation methods (Hsieh & Chao, 2017, 16,004 reads for natural environment and 55,205 reads for feeding trial consumers). We rarefied using the rrarefy() function in the vegan (version 2.5.6) package in R and rarefied the field and laboratory consumers separately.

We then selected all ASVs that matched potential diet items for the natural environment consumers (diet filtered to include all ASVs in the Kingdom: Animalia; Clade: Bilateria, excluding consumer DNA) and just the offered diet item for the feeding trial consumers (including species: *Oxya japonica*, genus: *Oxya*, and family: Acrididae, excluding those which only matched to order). Because the consumer species *H. venatoria* is the only species in the family Sparassidae on Palmyra Atoll, removing consumer DNA meant excluding all ASVs that received a family‐level taxonomic assignment of “Sparassidae.” As all ASVs received family‐level taxonomic assignment, we pooled ASVs that matched at the family level into one taxonomic unit using cumulative read abundance (i.e., all ASVs matched to *diet family A* were pooled into *diet family A* taxonomic unit), a practice common in diet metabarcoding (Kartzinel et al., 2015) and predator–prey interaction (Brose et al., 2019) studies.

### 2.8 Statistical analyses

For potential diet detection and rarefied abundance in both sets of consumers (natural environment and feeding trial), we used generalized linear models to assess the effect of surface sterilization treatment. For prey detection, we used all potential (natural environment) or offered (feeding trial) diet item detection (presence–absence per sample) as the response variable in the full model with surface sterilization as a fixed effect and a binomial distribution. For rarefied diet abundance, we only assessed consumers for which we had detected diet and not those with no diet detection (*n* = 33 of 37 for natural environment; *n* = 14 of 19 for feeding trials). For this model, we treated the number of all potential (natural environment) or offered (feeding trials) diet DNA reads per sample as the response variable, surface sterilization treatment as a fixed effect, total read abundance of the sample (constant across all) as an offset term, and a Poisson or negative binomial distribution (to correct for overdispersion when needed). We assessed differences in per‐sample potential diet richness among sterilization treatments for the natural environment consumers using generalized linear models with the number of potential diet items per sample as the response variable (both family‐level taxonomic units or ASVs), surface sterilization treatment as the fixed effect and a Poisson or negative binomial distribution (to correct for overdispersion when needed). We assessed differences in potential diet item composition with family‐level taxonomic units between surface sterilized and unsterilized consumers using a presence–absence PERMANOVA model fit with a binomial mixed effects model with surface sterilization treatment as a fixed effect, a random intercept term for potential diet item, and a random slope term for surface sterilization treatment. We also assessed ASV composition as a representation of potential prey composition using a canonical correspondence analysis (CCA) with surface sterilization as a predictor variable. We performed these analyses along with multiple other supplementary analyses and approaches, which can be found in the Supplementary Information (Appendices S4 and S5).

For all generalized linear models and mixed models, we performed model selection by comparing the full model (including the fixed effect of surface sterilization treatment) to a null model without this effect. All models were called in the glmmTMB package (version 1.0.0, Brooks et al., 2017) in R (version 3.6.1) We chose the best‐fitting model based on size‐corrected AIC values (MuMIn package version 1.43.15). For responses for which the best model included the surface sterilization treatment term, we examined the model summary to determine the standardized coefficients (*β*) and *p*‐value of the significance between marginal means of the levels of the surface sterilization fixed effect. We assessed model fit using diagnostics in the DHARMa package (version 0.2.7), including tests for heteroscedasticity, and for count models (Poisson or negative binomial), zero inflation and overdispersion (Bolker et al., 2009; Zuur et al., 2009). We performed the CCA using the vegan package in R, comparing a model with surface sterilization as a fixed effect to a null model using an ANOVA. All raw data, data cleaning, and data analyses are available online (Miller‐ter Kuile, 2020a, 2020b), and model outputs for primary and supplemental models can be found in Appendices S3 and S4.

## 3 RESULTS

### 3.1 PCR success, sequence merging, filtering, and clustering with UNOISE3 and DADA2

We successfully extracted DNA from 100% of samples (*n* = 72). Amplification success across all samples was 78%, with 56 of 72 initially extracted samples successfully amplified and were thus sequenced (Table 1). Seventy‐three percent (128 of 176) of ASVs matched to a taxonomic assignment. Twenty‐three percent of the total ASVs corresponded to potential diet items (41 of 176), and eight percent (14 of 176) corresponded to consumer DNA (the remaining 73 ASVs corresponded to nondiet items, including fungi, bacteria, and human DNA). Amplicon sequence variants that matched to the consumer comprised the majority of each sample (98 ± 0.6% of rarefied abundance compared to 1.5 ± 0.6% for potential diet and 0.3 ± 0.1% for nondiet). Eighty‐five percent of the potential diet ASVs received a species‐level taxonomic assignment (35 of 41) from either BLAST or BOLD taxonomic assignments, and every potential diet species received a family‐level and order‐level taxonomic assignment. In MEGAN, the family‐level assignments corresponded to 100% coverage results suggesting evidence of no mitochondrial pseudogenes (NUMTs) at the family level (Saitoh et al., 2016). There were no conflicting taxonomic assignments at the family level or higher between the BOLD and BLAST assignments.

### 3.2 Detection of potential diet items

**FIGURE 1:**
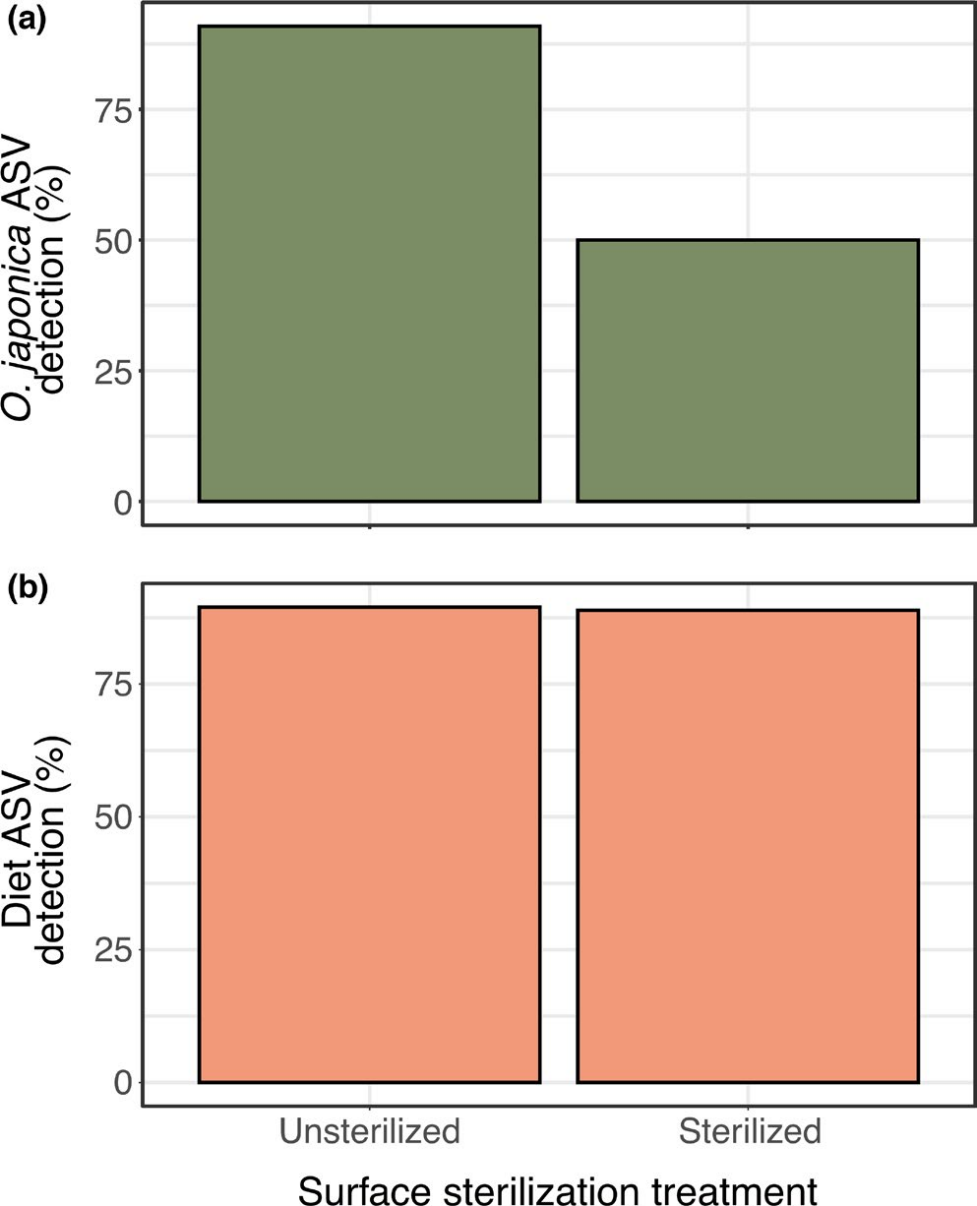
(a) Detection of all potential diet DNA in natural environment consumers that were and were not surface sterilized. Detection of diet DNA did not change with sterilization treatment. (b) Detection of offered diet (*Oxya japonica*) DNA in feeding trial consumers that were and were not surface sterilized. While the best‐fitting model based on AICc values indicated an effect of surface sterilization treatment (a decrease from 91% without surface sterilization to 50% with surface sterilization), the effect of this term in the model was statistically unclear (*p*‐value = .07)

We detected potential diet in 89% (33 of 37) of natural environment consumers and the offered diet in 74% (14 of 19) of feeding trial consumers. For natural environment consumers, family‐level taxonomic units corresponded to 20 families of potential diet items. The best model for potential diet detection in natural environment consumers was the null model that did not include surface sterilization treatment as a fixed effect (Figure 1, Appendix S4). For feeding trial consumers, one ASV matched to the offered diet (species: *O*. *japonica*, genus: *Oxya*, and family: Acrididae), and the best model for diet detection included the fixed effect of surface sterilization treatment, though the model without the surface sterilization term was within two AICc values (ΔAICc = 1.59) and the effect of the surface sterilization term was not statistically clear (*β* = −2.3; *p*‐value = .07). We detected offered prey in 50% of consumers that had been surface sterilized compared to 91% of those consumers that were not surface sterilized.

### 3.3 Proportion of potential diet DNA

**FIGURE 2:**
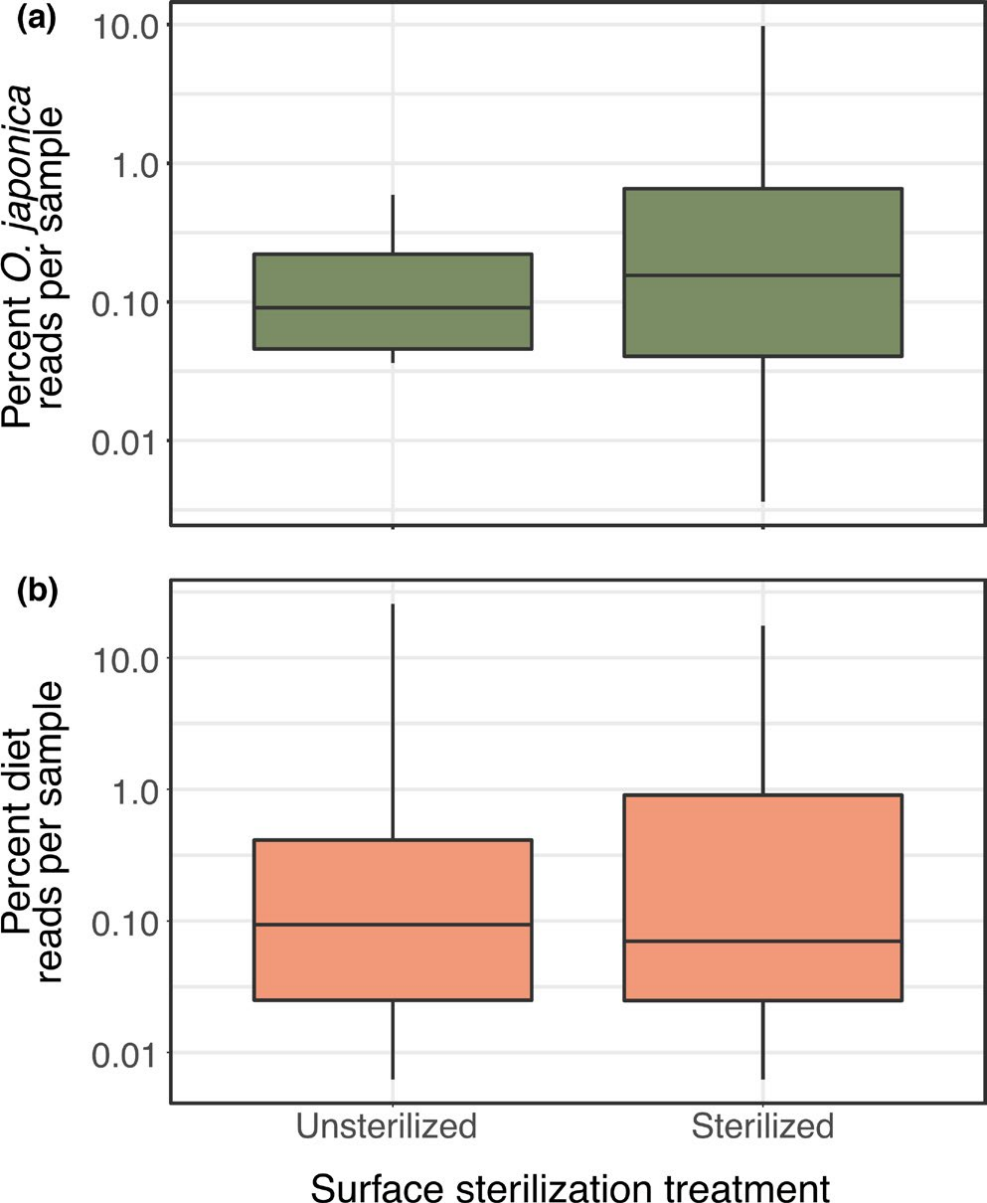
Neither the (a) proportion of total potential diet DNA in natural environment consumers or the (b) proportion of offered diet item DNA in feeding trial consumers significantly changed with surface sterilization treatment

For natural environment consumers, potential diet rarefied DNA sequence reads represented 2.0% (±1.0%) of total per‐sample DNA sequence abundance (Figure 2). In feeding trial consumers, offered diet DNA sequence reads represented 0.8% (±0.7% *SE*) of total per‐sample DNA sequence abundance. For both natural environment and feeding trial consumers, the null models that did not include surface sterilization treatment as a fixed effect were the best models of diet DNA read abundance.

### 3.4 Potential diet richness and composition in natural environment consumers

**FIGURE 3:**
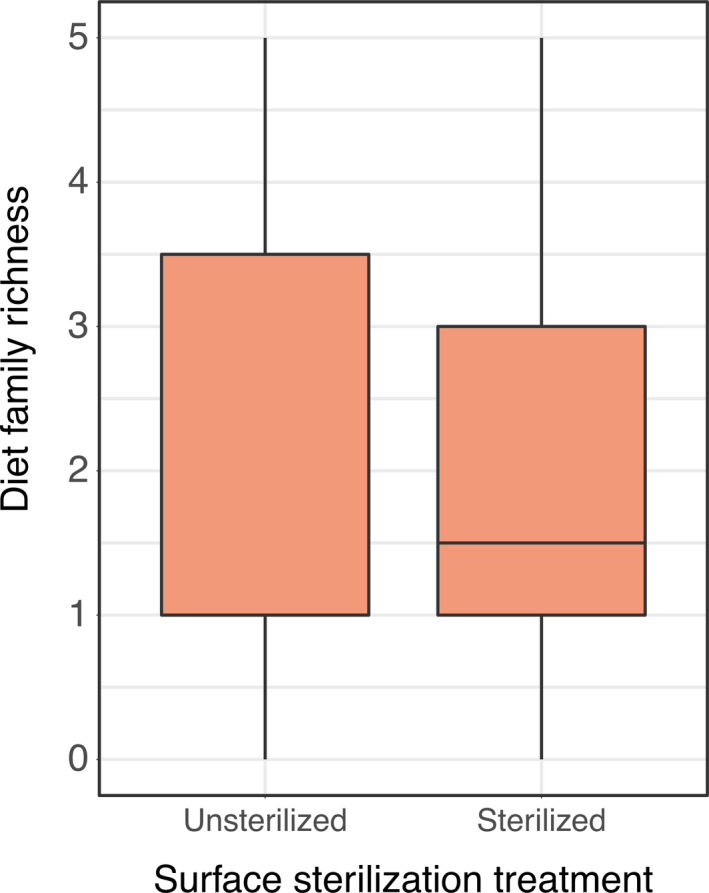
In natural environment consumers, surface sterilization did not alter per‐sample diet richness of either family‐level or ASV‐level taxonomic units

**FIGURE 4:**
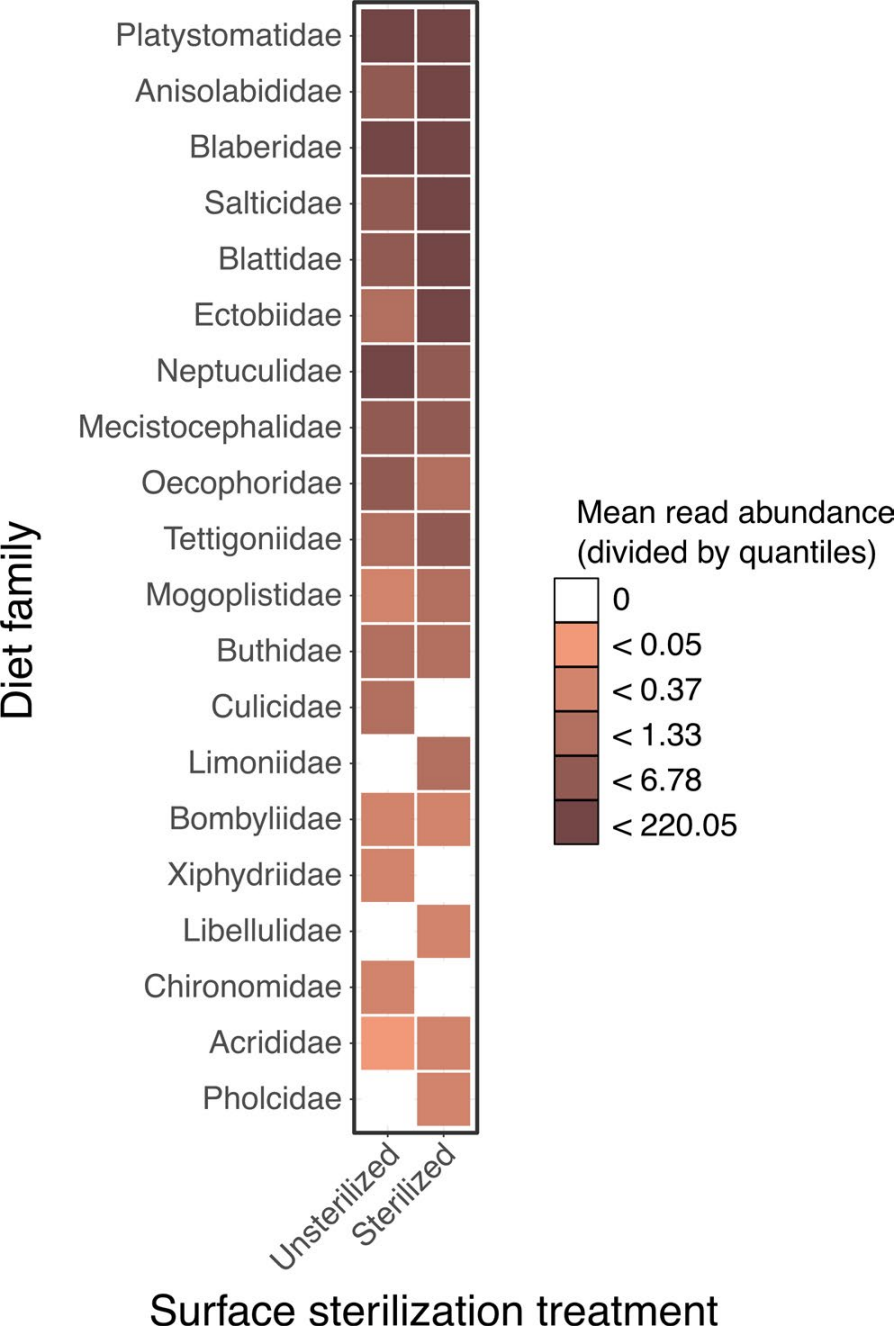
For natural environment consumers, surface sterilization did not alter the composition (either with a presence–absence of abundance model) of potential diet items of either family‐level taxonomic units or ASV‐level taxonomic units. In this figure of family‐level taxonomic units by surface sterilization treatment, presence is indicated by a colored box and abundance is indicated by color depth (divided by quartiles due to wide variation in DNA sequence abundance)

For family‐level taxonomic units, potential diet richness per natural environment consumer was an average 2.08 (±0.26 *SE*) families per individual sample, with a maximum of 5 diet families in one consumer diet (Figure 3). Richness of potential diet ASVs for these consumers was similar, with an average of 2.32 (±0.31) potential diet ASVs per sample with a maximum of 7 ASVs in one consumer (Figure 3). The best models for per‐sample potential diet richness for both family‐level taxonomic units and ASV‐level, as well as both family‐level PERMANOVA and ASV‐level CCA, were the null models which did not include surface sterilization treatment as a predictor (Figure 4, Figure S1). Diet families came from insect, arachnid, and centipede orders (insects: Diptera (5), Dermaptera (1), Blattodea (3), Lepidoptera (3), Orthopotera (3), Hymenoptera (1), Odonata (1); Arachnids: Araneae (2); Scorpiones (1); and Centipedes: Geophilomorpha (1), Figure 4).

## 4 DISCUSSION

Surface sterilization does not change diet measures in diet DNA metabarcoding data for the predatory consumer *H. venatoria* in either natural settings or a feeding trial environment, suggesting that surface sterilization is not a necessary step for this consumer. Our results suggest that various measures of diet, including potential diet detection, rarefied abundance, richness, and composition, are not significantly altered by surface sterilizing consumers prior to DNA metabarcoding. For potential diet richness and composition, in particular, these results did not change when considering potential diet in combined family‐level taxonomic units (making them comparable with food web studies in this field, e.g., Brose et al., 2019) and when considering richness of molecular taxonomic units (ASVs). We detected diet across 84% of the total consumers in our study (*n* = 47 of 56), including 20 diet families. Diet DNA metabarcoding has high potential to contribute diet information for small consumers with cryptic feeding habits. Furthermore, it appears that current protocols that do not include surface sterilization steps are sufficient to determine potential diet for these consumers.

The field of diet DNA metabarcoding has not universally adopted surface sterilization practices into common protocols, in particular for studies including DNA extraction of full organisms or body parts without dissection (e.g., Jacobsen et al., 2018; Wirta et al., 2014). We demonstrate that surface sterilization does not seem necessary to avoid contamination effects. The evident lack of the effects of surface contaminants in our study contrasts with obvious surface contaminants that alter ecological interpretations in other fields using high‐throughput sequencing to determine community diversity, particularly fungal endophyte studies (Burgdorf et al., 2014). One reason for this difference may be that fungal spores are widespread on and in the surfaces of most environments and organisms (Després et al., 2012) and likely to contaminate studies targeting specific subgroups of these communities. Indeed, even in our dataset, some sequences matched to fungal taxonomies. The fact that these nontarget sequences did not alter our DNA metabarcoding data by hiding target diet DNA, even with the relative rarity of diet DNA compared to consumer DNA (0.006%–26% of each sample), is likely due to differences in biomass of these sources of DNA in our samples and the specificity of our DNA size‐selection protocol and PCR primers (Elbrecht et al., 2017; Krehenwinkel et al., 2017). Therefore, our results are promising both in validating the robustness of findings from past diet DNA studies that have not implemented surface sterilization treatments, but also highlight that diet DNA metabarcoding using broad, universal primer sets (e.g., those in this study) is an effective tool even when DNA sequence data contain potential environmental contaminants (Appendix S5, Figure S5).

While we saw no widespread support of the necessity for surface sterilization in our study, a model from the feeding trial that includes surface sterilization performed slightly better than one without this treatment (ΔAICc = 1.59). Thus, it is possible that contained environments may be more prone to contamination than open terrestrial environments. We see this result as an ideal starting point for next steps in validating diet DNA metabarcoding in similar contexts. Specifically, because this study had a relatively limited sample size (*n* = 8 and 11 in each sterilization treatment group) and because we did not confirm ingestion, a similar trial including crossed treatments of sterilization with different forms of diet item contact (e.g., Greenstone et al., 2012) would provide additional evidence of the effects of surface sterilization or surface contamination. Further exploration of these results might reveal that the decision to surface sterilize prior to diet DNA metabarcoding may matter more in some environments and experiments than others (e.g., where diet items are in high density or consumers have long handling times (Abrams & Ginzburg, 2000; Samu & Biro, 1993). Furthermore, as earlier studies targeting particular consumer diet pairs explored (e.g., Greenstone et al., 2012), the field of diet DNA metabarcoding is ripe for a comparison of surface sterilization techniques.

Diet DNA metabarcoding can empirically provide diet descriptions for a suite of consumers important to food web ecology and the maintenance of biodiversity on the planet (Stork, 2018).

Characterizing consumptive interactions for small, cryptic species for the first time will build a better picture of nature's complexity and allow ecologists to confidently query how species interactions will change with continued anthropogenic disturbance (Tylianakis et al., 2008). Like any method for determining consumptive interactions in nature, DNA metabarcoding continues to be refined, especially as tools and data emerge (Krehenwinkel et al., 2019; Kvist, 2013). This study builds on past efforts to refine diet DNA metabarcoding by using surface sterilization to pinpoint potential sources of error in diet DNA data. Here, we found that, on the whole, surface sterilization seems unnecessary in two contexts (terrestrial environments and contained feeding trials) when extracting DNA from body parts of invertebrate taxa. Continued context‐specific refinement of surface sterilization protocols, and of other steps in diet DNA metabarcoding, will improve the widespread utility of diet DNA metabarcoding across consumer groups and environments.

## CONFLICT OF INTEREST

None declared.

## AUTHOR CONTRIBUTIONS

**Ana Miller‐ter Kuile:** Conceptualization (equal); data curation (lead); formal analysis (lead); funding acquisition (equal); investigation (lead); methodology (equal); project administration (lead); visualization (lead); writing–original draft (lead); writing–review and editing (lead). **Austen Apigo:** Conceptualization (equal); formal analysis (supporting); investigation (supporting); methodology (equal); project administration (supporting); visualization (supporting); writing–original draft (supporting); writing–review and editing (supporting). **Hillary S. Young:** Conceptualization (supporting); formal analysis (supporting); funding acquisition (equal); investigation (supporting); project administration (supporting); supervision (lead); writing–original draft (supporting); writing–review and editing (supporting).

## Supporting information

Appendix S1‐S5

Appendix S6

## Data Availability

Raw sequence data are available on GenBank (BioProject: PRJNA639981). Cleaned sequence data and analyses are available on Dryad (DOI: https://doi.org/10.5061/dryad.gqnk98snc).
