# Supplementary material for: Effects of consumer surface sterilization on diet DNA metabarcoding data of terrestrial invertebrates in natural environments and feeding trials
Source: Ecol Evol. 2021 Aug 9;11(17):12025–34. doi: 10.1002/ece3.7968 (PMC8427582; doi:10.1002/ece3.7968)
Supplement: Supplementary file 1 — Appendix S1‐S5 [file ECE3-11-12025-s002.docx]

**Supplementary Information**

Title: Effects of surface sterilization on diet DNA metabarcoding data of invertebrate consumers in mesocosm and natural environments

Authors: Ana Miller-ter Kuile, Austen Apigo, Hillary S. Young

Table of Contents

[Appendix S1: Comparisons between UNOISE3 and DADA2 with and without BBSplit 3](#_Toc58313365)

[Motivation 3](#_Toc58313366)

[The data 4](#_Toc58313367)

[Outline of pipeline performance 5](#_Toc58313368)

[Pipeline performance 6](#_Toc58313369)

[A. Clustering specificity (positive and negative control ASVs) 6](#_Toc58313370)

[B. Total ASVs (richness) 8](#_Toc58313371)

[C. Number of prey ASVs (diet richness) 11](#_Toc58313372)

[D. Phylogenetic diversity of prey ASVs 13](#_Toc58313373)

[E. Prey read abundance 14](#_Toc58313374)

[F. Percent of ASVs that are prey 16](#_Toc58313375)

[A. Percent of reads that are prey 17](#_Toc58313376)

[H. Amount of known diet reads 18](#_Toc58313377)

[Pipeline Performance Summary 20](#_Toc58313378)

[Appendix S2: BBSplit Methods 23](#_Toc58313379)

[Appendix S3: Model outputs for GLMMs 25](#_Toc58313380)

[1. Prey Detection 25](#_Toc58313381)

[A. Mesocosm 25](#_Toc58313382)

[B. Natural 28](#_Toc58313383)

[C. Summary 32](#_Toc58313384)

[2. Prey DNA Abundance 32](#_Toc58313385)

[A. Mesocosm 32](#_Toc58313386)

[B. Natural 37](#_Toc58313387)

[C. Summary 43](#_Toc58313388)

[3. Natural: Prey DNA Richness and Composition 43](#_Toc58313389)

[A. Richness 43](#_Toc58313390)

[B. Composition 48](#_Toc58313391)

[C. Summary 52](#_Toc58313392)

[Appendix S4: Model outputs for supplementary data analyses 54](#_Toc58313393)

[1. DNA abundances 54](#_Toc58313394)

[A. Mesocosm all prey 54](#_Toc58313395)

[B. Mesocosm non-diet 58](#_Toc58313396)

[D. Summary 68](#_Toc58313397)

[2. Natural abundance-based diet composition 69](#_Toc58313398)

[3. Mesocosm all prey composition 74](#_Toc58313399)

[A. Presence-based composition 74](#_Toc58313400)

[A. Abundance-based composition 75](#_Toc58313401)

[Appendix S5: Supplementary Figures 77](#_Toc58313402)

[1. DNA cleaning with Ampure XP beads 77](#_Toc58313403)

[2. Library preparation schematic 78](#_Toc58313404)

[3. Denoising algorithm process 79](#_Toc58313405)

[4. Sequencing depth 80](#_Toc58313406)

[5. All DNA source abundances 81](#_Toc58313407)

[6. Mesocosm consumer presence and abundance effect sizes 84](#_Toc58313408)

[7. Natural consumer presence and abundance effect sizes 85](#_Toc58313409)

[8. Natural consumer presence and abundance of prey items by individual 86](#_Toc58313410)

[9. Natural consumer presence and abundance of prey ASVs 86](#_Toc58313411)

# Appendix S1: Comparisons between UNOISE3 and DADA2 with and without BBSplit

## Motivation

This project creates a workflow for analyzing the diets of invertebrate predators using high throughput sequencing of gut contents. This method provides a promising way to get highly-resolved diet data from consumers across ecosystems.

**Bioinformatics challenge**: To extract all possible prey items from predator guts when predator and prey are taxonomically similar, it is best practice to use a set of PCR primers that target all possible prey. However, a side effect of this is that these primers will also end up amplifying a large amount of predator DNA. As a result, these datasets are dominated by predator DNA, and so detecting relatively rare prey sequences in these datasets is key. As the molecular ecology field moves toward using amplicon sequence variants (ASVs) as biologically-real units of biodiversity in high throughput datasets, these types of datasets dominated by predator DNA are even more challenging since these ASV clustering pipelines use sequence abundance as a way to cluster sequences into similar, biologically-real groups of sequences. Therefore, any clustering pipeline used for DNA diet data dominated by the predator must

- detect prey sequences that are taxonomically similar to the DNA of predators and
- detect prey sequences that are relatively rare compared to the DNA of predators


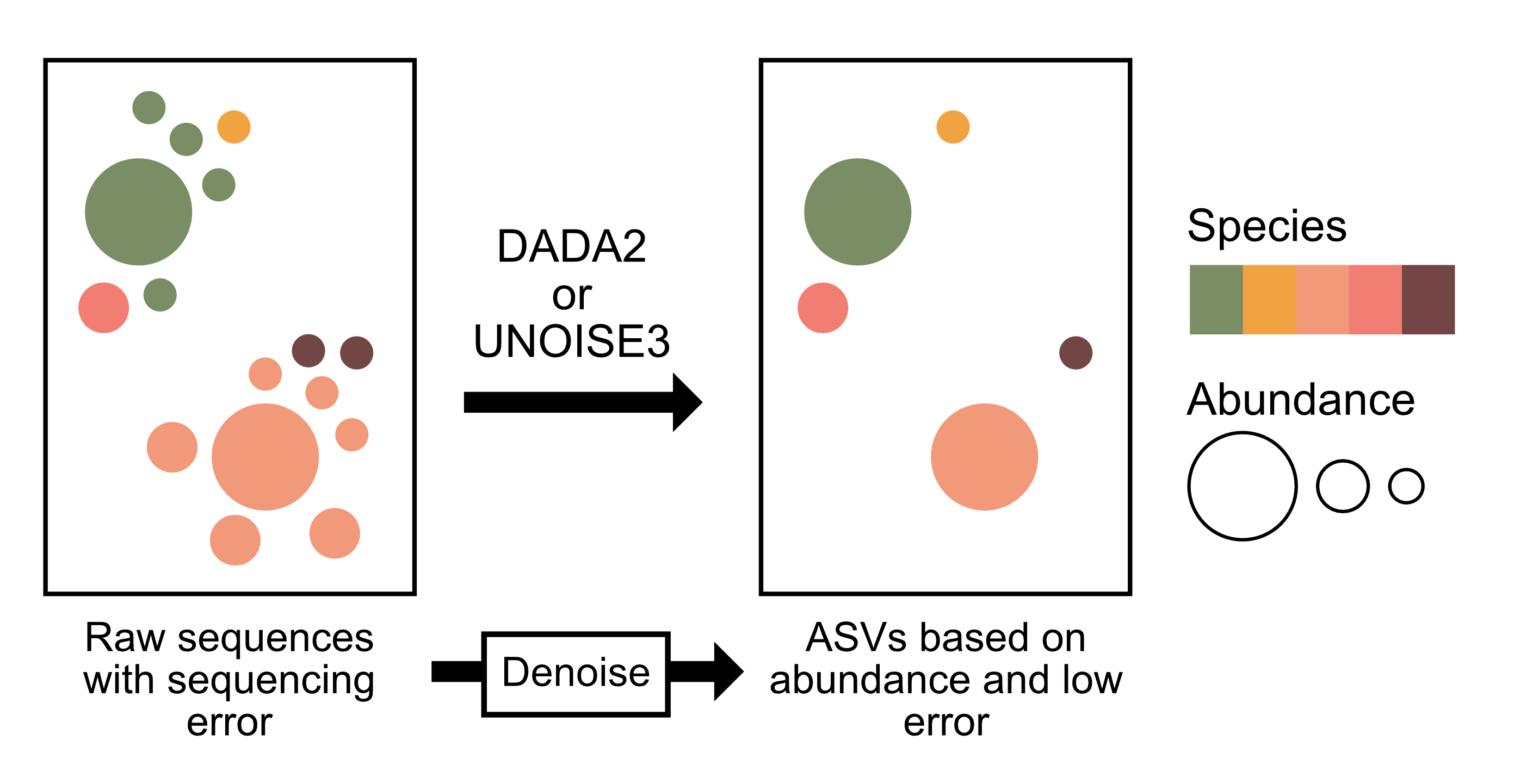


Figure 1: Denoising pipelines (including DADA2 and UNOISE3) consider sequence abundance and error rate in raw sequence datasets when removing sequences that have high error and low abundance and combining their reads with those of their most similar neighbor sequence with high abundance and low error (amplicon sequence variants or ASVs). Because our dataset has high abundance predator DNA with lower abundance prey DNA, any denoising pipeline we use must accurately be able to distinguish low abundance prey ASVs from high abundance predator ASVs.

This supplement assesses how different denoising/clustering pipelines perform with these types of datasets and provide a template for other studies interested in interactions between invertebrate predators and prey, but with the idea that the same sort of process could work well for other study systems (i.e. vertebrates that eat vertebrates) as well.

## The data

The data for this project consists of 56 individuals of the spider species *Heteropoda venatoria* from Palmyra Atoll National Wildlife Refuge. We collected 37 of these individuals in the field in 2015 and immediately froze them in a -80C freezer until processing in 2019. The remaining 19 individuals we collected in 2017, starved in mesocosms in the lab for 24 hours, and then fed a known diet item (the grasshopper *Oxya japonica*). After spiders had been in mesocosms with prey items and eaten them (12-24 hours), we froze them at -20C. Prior to final preservation in 80% EtOH, we surface sterilized 8 of these 19 individuals by submerging them in a 10% bleach to DI water solution for 2 minutes and then washing them in DI water for 2 minutes. We performed the same sterilization protocol on 18 of the 37 field-collected individuals after we had brought them to the lab from the field (in 2019). (see Methods document for full extraction, PCR, and sequencing protocols).


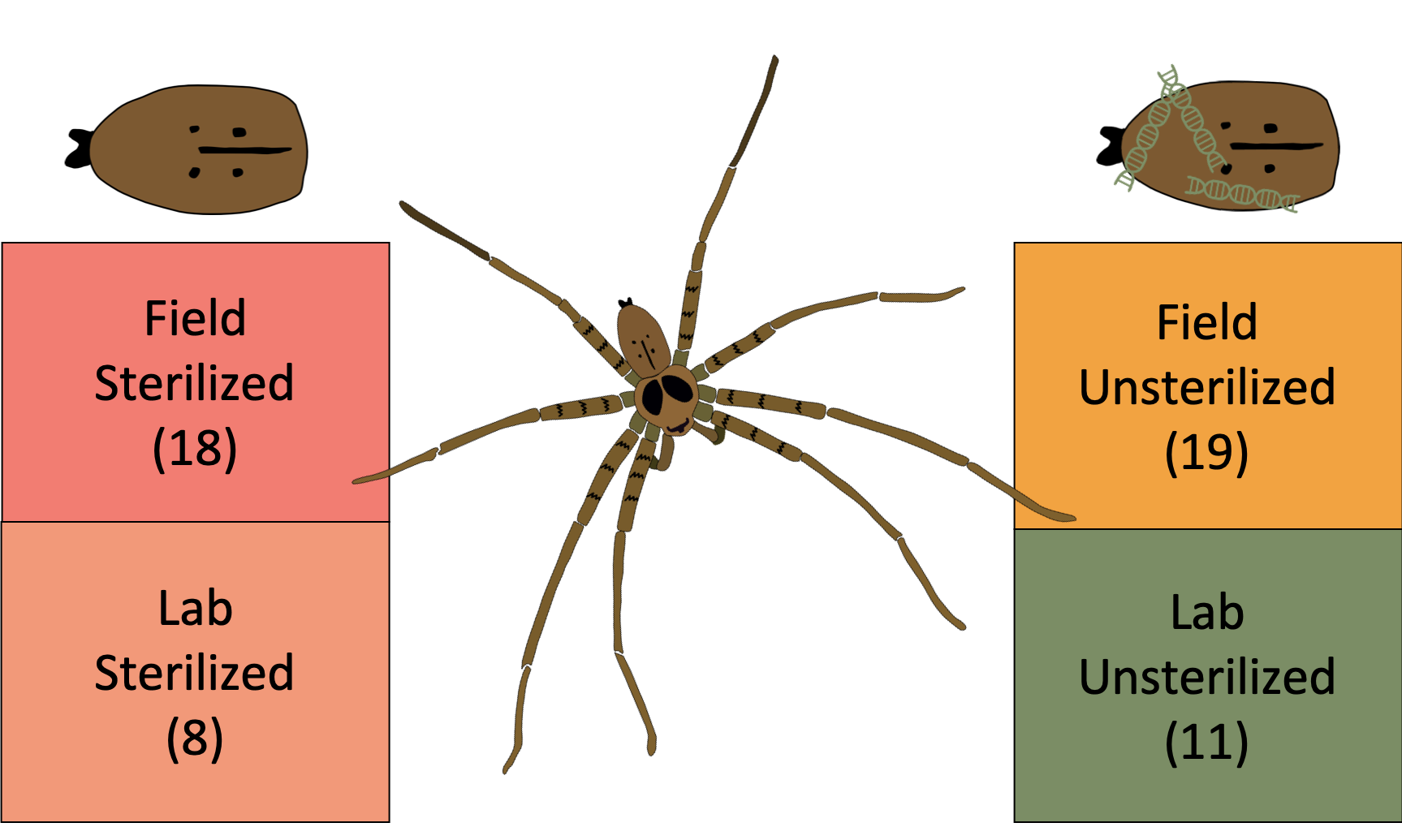


Figure 3: Our surface-contamination experiment included four groups of varying sample sizes: both field-collected and lab-fed sterilized individuals and unsterilized individuals.

We ran all samples on an Illumina MiSeq platform at the UCSB Genetics Core. We cleaned low-quality sequences from this set of ~15 million sequences returned from the genetics core, and then used two clustering pipelines (DADA2 and UNOISE3) that use sequence abundance and quality to combine similar sequences (which are often different because of errors in amplification or sequencing and actually represent the same sequence) into clusters representing the most common real sequence. We performed each of these clustering pipelines twice: once on the complete list of sequences, and once on a set of sequences that had been “cleaned” of predator and non-diet sequences using the program BBSplit. After the denoising/clustering process, we are left with a matrix very similar to any community matrix, with columns corresponding to each sample (spider individual) and rows corresponding to ASVs (sequences) in that individual. In this way, each spider becomes functionally a “community” of species which can be treated very similarly to any other matrix of community data (i.e. in vegan with multidimensional analyses, etc).

## X HEV.100_S22 HEV.101_S23 HEV.102_S1 HEV.103_S2 HEV.104_S3 HEV.105_S4
## 1 ASV_1 177549 219335 186660 199570 134902 145046
## 2 ASV_2 0 0 0 0 0 0
## 3 ASV_3 0 0 0 0 0 0
## 4 ASV_4 521 0 0 0 15393 0
## 5 ASV_5 5 0 0 0 0 0
## 6 ASV_6 0 0 0 0 0 0
## 7 ASV_7 0 0 32 0 0 0
## 8 ASV_8 0 0 0 80 0 8
## 9 ASV_9 0 0 0 0 16 0
## 10 ASV_10 0 0 0 0 0 0

## Outline of pipeline performance

- *Clustering specificity*: Positive and negative controls map to fewer ASVs
- *Total ASVs (richness)*: A greater number of ASVs means a higher likelihood the full diversity of the sample has been detected
- *Number of prey ASVs (diet richness)*: A higher diet richness means the pipeline better distinguishes prey ASVs from predator
- *Phylogenetic diversity of prey ASVs*: A better pipeline will detect a broader phylogenetic diversity of prey
- *Prey read abundance*: More prey reads means a higher likelihood of picking up rarer prey items
- *Percent of ASVs that are prey*: This corrects for variable sequencing depth and a higher percentage means a higher likelihood that all prey are detected
- *Percent of reads that are prey*: Again, correcting for sequencing depth, a higher percentage means a higher likelihood that all prey are detected
- *Amount of known diet reads*: A better pipeline will be better at detecting a diet item we KNOW to be present in all samples

## Pipeline performance

#non-zero reads should only be in the positive control samples:
u3_pos_ct <- u3_uc %>%
 filter(ASV %in% c("Zotu4", "Zotu2", "Zotu3")) %>%
 gather(sample, reads, CL1:QC1) %>%
 filter(reads > 0) %>%
 mutate(type = "u3")

############################
#Justify UNOISE vs. DADA2####
############################
#also, in justifying UNOISE, want to check that the positives got more reads
#NOTE: PUT THIS IN THE PIPELINE COMPARISON SCRIPT!!!!####
d2 <- read.csv(here("data", "denoised_data", "ASV_tables",
 "dada2_uc_asv_tab.tsv"), sep = "\t")

colnames(d2) <- sapply(str_split(colnames(d2), "_"), function(x){return(x[[1]])})
d2 <- rename(d2, "ASV" = "X")

d2_pos_cont <- d2_uc %>%
 filter(ASV %in% c("ASV_10", "ASV_2", "ASV_16")) %>%
 gather(sample, reads, CL1:QC1) %>%
 filter(reads > 0) %>%
 mutate(type = "d2")

positive <- u3_pos_ct %>%
 bind_rows(d2_pos_cont) %>%
 group_by(sample, type) %>%
 summarise(reads = sum(reads))

pos_reads <- positive %>%
 group_by(type) %>%
 summarise(mean = mean(reads), sd =sd(reads), total = n(), se = sd/sqrt(total)) %>%
 ggplot(aes(x = type, y = mean)) +
 geom_bar(stat = "identity", position = "dodge") +
 theme_bw() +
 labs(x = "Pipeline", y ="Average reads per positive control") +
 geom_errorbar(aes(ymin = mean -se, ymax = mean+se))

We looked at a bunch of different ways the pipelines performed both bioinformatically and ecologically. If you don’t want to spend time on each one, you can jump to the pipeline performance [summary table](#pipelines), which provides the scores of each pipeline in each measure of performance.

### A. Clustering specificity (positive and negative control ASVs)

Different clustering methods may have more or less specificity in their clustering. We checked for this by running several positive control samples and one negative control sample and looking at how different clustering pipelines assigned ASVs to these controls. The positive controls are cloned fungal species which Austen uses in his work. Because they are clones, they should map to one or very few ASVs. The negative controls were samples we ran through PCRs with no DNA in them and quantified to zero before submitting. Therefore, these should map to zero ASVs. **Takeaway**: smaller is better for both positive and negative control ASV counts.


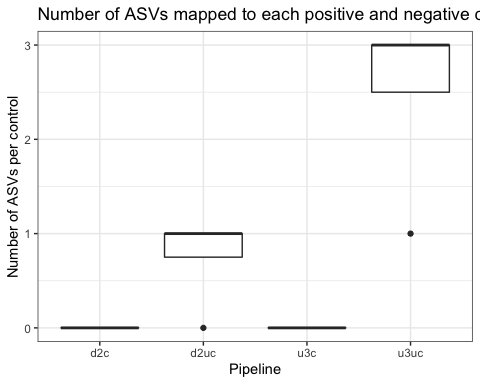


It seems that, first of all, cleaning removes all ASVs for the controls, which we would expect, since we filtered these based on known prey and predator ASVs. Furthermore, it seems that DADA2 does a better job of assigning fewer ASVs to positive controls than UNOISE3. Furthermore, the negative control had zero reads assigned to any reads in DADA2, while UNOISE3 assigned one ASV a value of 1 read for the negative control.

On the other hand, when we look at how many sequence reads each control received (indicative of the pipeline to match DNA to denoised clusters), it looks like UNOISE3 does a much better job on average of mapping more sequence reads to positive controls:


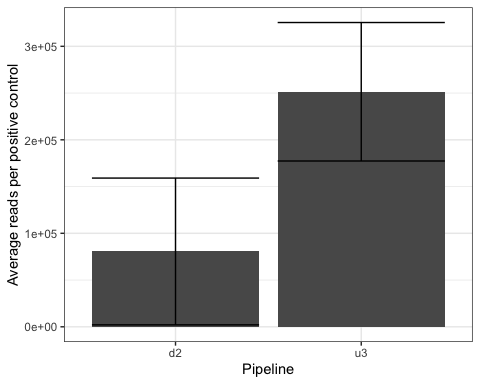


**Winner: UNOISE uncleaned**

### B. Total ASVs (richness)

A good clustering pipeline will be able to pick up all the diversity in a dataset. In this case, a higher number of total ASVs (roughly, species richness) means better clustering method, since we want to capture the greatest diversity possible. However, we will go into some more specific measures of richness (prey richness) next, since just the total number of ASVs may not actually be prey richness, but may instead be the pipeline clustering more predator or non-prey ASVs.

We looked at both total ASVs produced by each pipeline as well as the number of ASVs each clustering pipeline assigned to each sample.


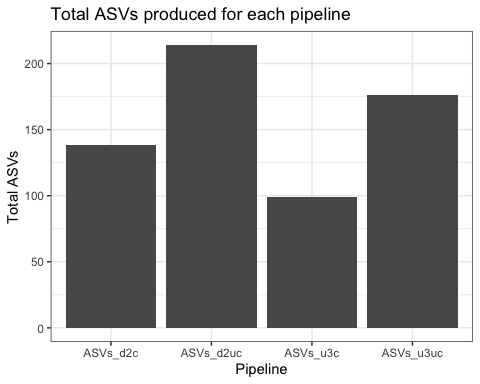


The uncleaned datasets produce more ASVs, and DADA2 produces more total ASVs than UNOISE3.

**Winner: DADA2**

When we look at the total number of ASVs produced per sample, we see a different pattern, however:


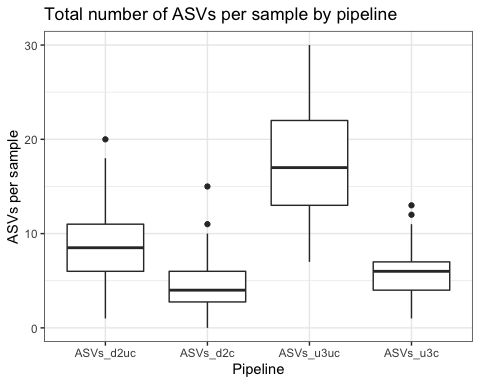


Indeed, these differences among pipelines are statistically significant based on a random effects model with a full model structure of (comparing it to a null without pipeline, and then estimating marginal means between each pair):

tot_ASV_mod <- glmmTMB(value ~ pipeline + (1|sample),
 data = by_sample_long,
 family = "genpois")

With significant pairwise differences between all pipelines:

## contrast estimate SE df t.ratio p.value
## ASVs_d2uc - ASVs_d2c 0.615 0.0593 218 10.367 <.0001
## ASVs_d2uc - ASVs_u3uc -0.677 0.0434 218 -15.595 <.0001
## ASVs_d2uc - ASVs_u3c 0.431 0.0564 218 7.644 <.0001
## ASVs_d2c - ASVs_u3uc -1.292 0.0540 218 -23.921 <.0001
## ASVs_d2c - ASVs_u3c -0.184 0.0648 218 -2.839 0.0254
## ASVs_u3uc - ASVs_u3c 1.108 0.0507 218 21.881 <.0001
##
## Results are given on the log (not the response) scale.
## P value adjustment: tukey method for comparing a family of 4 estimates


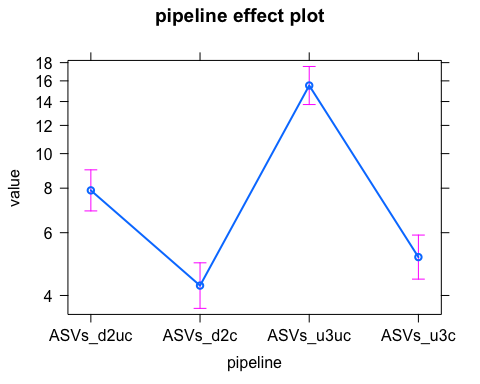


The takeaway here is that both UNOISE3 unclean and clean produce more ASVs than their DADA2 counterparts.

**Winner: UNOISE3 uncleaned and cleaned**

### C. Number of prey ASVs (diet richness)

A higher total number of ASVs does not necessarily mean that a clustering pipeline is the best option, since these newly discovered ASVs may not actually be ASVs of interest, but rather ASVs of predators or other non-prey items (contamination, for example). Therefore, a better measure of whether a pipeline works best for the purposes of this study is to look at how well it clusters prey ASVs. We did this by combining taxonomic data to our ASV list, and assigning broad taxonomic categories to these ASVs. We combined taxonomic data from both GenBank (using BLAST and MEGAN) and from the BOLD IDEngine database. We combined taxonomic assignments from both sources and removed any assignment which did not match between these two databases. Anything that mapped to the predator Family, Genus, or Species was given a taxonomic category of “predator”, anything that could possibly be prey (including arthropods and vertebrates in this dataset) were given a taxonomic category of “prey”. Anything that was not given a specific taxonomic assignment but which could have been prey or predator was given the category of “unknown” (i.e. a BLAST taxonomic assignment of “Arthropoda”). There were other items in the dataset that were definitely not predators or prey (mostly fungi) and these were given a taxonomic assignment of “non-prey”. Our final category included any ASV that was clustered but which was not assigned a taxonomy; this category was “no hit”.

We then subset just the prey ASVs from this dataset and compared the per sample number of prey ASVs assigned by each pipeline, which is visualized here:


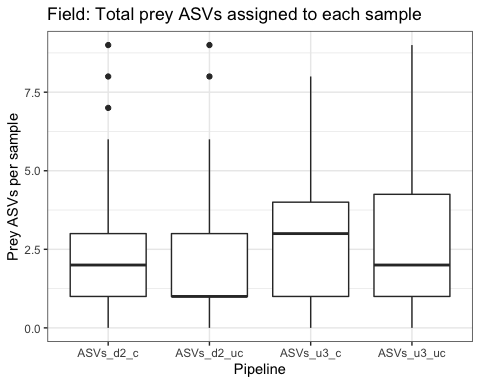


We again looked for significant across group differences in prey ASVs using a mixed model (comparing it to a null without pipeline, and then estimating marginal means between each pair):

lme_prey_ASVs <- glmmTMB(ASVs ~ pipeline + (1|sample),
 data = taxa_ASVs_prey,
 family = "genpois")

And looked at pairwise differences between these groups:

## contrast estimate SE df t.ratio p.value
## ASVs_d2_c - ASVs_d2_uc 0.1098 0.0707 218 1.552 0.4084
## ASVs_d2_c - ASVs_u3_c -0.2920 0.0677 218 -4.312 0.0001
## ASVs_d2_c - ASVs_u3_uc -0.3334 0.0675 218 -4.936 <.0001
## ASVs_d2_uc - ASVs_u3_c -0.4018 0.0699 218 -5.753 <.0001
## ASVs_d2_uc - ASVs_u3_uc -0.4432 0.0693 218 -6.399 <.0001
## ASVs_u3_c - ASVs_u3_uc -0.0414 0.0615 218 -0.672 0.9074
##
## Results are given on the log (not the response) scale.
## P value adjustment: tukey method for comparing a family of 4 estimates


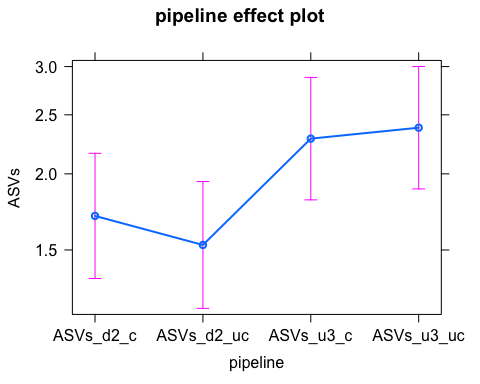


What we see is that cleaning does not significantly increase the number of ASVs assigned to prey items for either DADA2 or UNOISE3. However, what we see is that UNOISE3 assigns more prey ASVs to each sample, suggesting that UNOISE3 is better at detecting a greater prey richness than DADA2

**Winner: UNOISE unclean**

### D. Phylogenetic diversity of prey ASVs

Not only might one pipeline be better at detecting total prey richness, but might also be able to cluster a broader phylogenetic diversity of prey items. This might be especially important in systems where predators are generalists and feed on organisms across broad taxonomic groups. Because the number of ASVs assigned to prey per sample is fairly low (less than 10 per sample) and because we are interested in just detection here and not necessarily abundance variations among samples, we will just qualitatively assess Faith’s PD for each pipeline, summing the prey ASVs for each pipeline. Furthermore, we will combine phylogenetic analyses at the Order and Family level, since we have good taxonomic representation at these levels (15 orders, 19 families)

UNOISE3 unclean picks up the greatest phylogenetic diversity. It is important to note that this is in spite of the fact that order richness is greater in the DADA2 dataset. UNOISE3 unclean has the lowest phylogenetic diversity, which is probably because we trained the cleaning program with DADA2 and some of the phylogenetic diversity of the UNOISE3 unclean dataset was removed. Based on the phylogenetic tree, DADA2 picked up Lepidoptera (moths and butterflies) and Sarcoptiformes (mites), while UNOISE3 picked up Squamata (geckos).

Again, UNOISE3 unclean picks up the greatest phylogenetic diversity. It is important to note that this is in spite of the fact that, again, family richness is greater in the DADA2 dataset. UNOISE3 unclean has the lowest phylogenetic diversity, which is probably because we trained the cleaning program with DADA2 and some of the phylogenetic diversity of the UNOISE3 unclean dataset was removed. DADA2 picks up Formicidae (ants) and Suidasiidae (mites), while UNOISE3 picks up Xiphydridae (wasps) and Gekkoniidae (geckos).

**Winner: UNOISE unclean**

### E. Prey read abundance

Similar to the number of ASVs assigned to prey, the number of sequence reads assigned to each of these ASVs is important because rarer prey items are more likely to be detected the greater abundance each prey ASV is in each sample.

We can visualize the number of reads of each ASV in each sample:


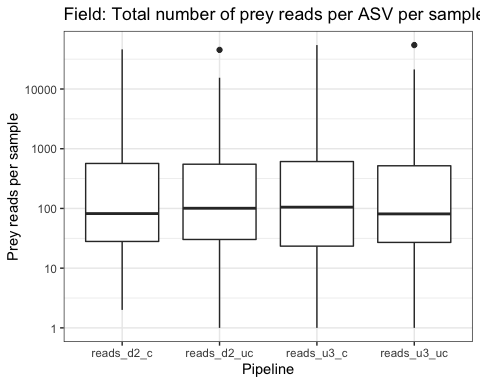


And again, we ran a mixed model to determine whether there are significant among pipeline detections in prey reads (comparing it to a null without pipeline, and then estimating marginal means between each pair):

read_mod <- glmmTMB(reads ~ pipeline + (1|sample),
 data = taxa_reads_prey,
 family = "genpois")

pairs(model.emm_preyreads)

## contrast estimate SE df t.ratio p.value
## reads_d2_c - reads_d2_uc 0.03215 0.0355 218 0.905 0.8021
## reads_d2_c - reads_u3_c -0.20844 0.0335 218 -6.221 <.0001
## reads_d2_c - reads_u3_uc -0.20328 0.0336 218 -6.052 <.0001
## reads_d2_uc - reads_u3_c -0.24060 0.0339 218 -7.089 <.0001
## reads_d2_uc - reads_u3_uc -0.23543 0.0339 218 -6.937 <.0001
## reads_u3_c - reads_u3_uc 0.00517 0.0317 218 0.163 0.9985
##
## Results are given on the log (not the response) scale.
## P value adjustment: tukey method for comparing a family of 4 estimates

Cleaning did not significantly increase prey read abundance. UNOISE3 produced more prey reads per ASV per sample than DADA2

**Winner: UNOISE3 unclean**

### F. Percent of ASVs that are prey

So far, we have been assuming that the total number of ASVs is independent of sequencing depth, which we know is not the case. Therefore, we ran the above ASV model again correcting for sequencing depth (using an offset of the total number of ASVs). These data can then be interpreted as the percent of all ASVs assigned to prey.


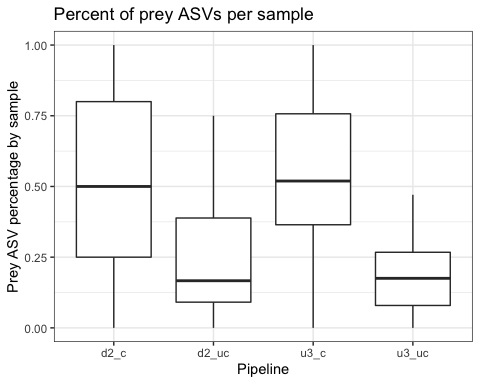


prey_prop_mod <- glmmTMB(prey ~ pipeline + (1|sample),
 data = ASV_totals_samples,
 family = "genpois",
 offset = log(tot))

## contrast estimate SE df t.ratio p.value
## d2_c - d2_uc 0.642 0.0661 215 9.719 <.0001
## d2_c - u3_c -0.118 0.0638 215 -1.851 0.2525
## d2_c - u3_uc 0.900 0.0631 215 14.249 <.0001
## d2_uc - u3_c -0.760 0.0638 215 -11.921 <.0001
## d2_uc - u3_uc 0.257 0.0629 215 4.090 0.0004
## u3_c - u3_uc 1.018 0.0562 215 18.106 <.0001
##
## Results are given on the log (not the response) scale.
## P value adjustment: tukey method for comparing a family of 4 estimates


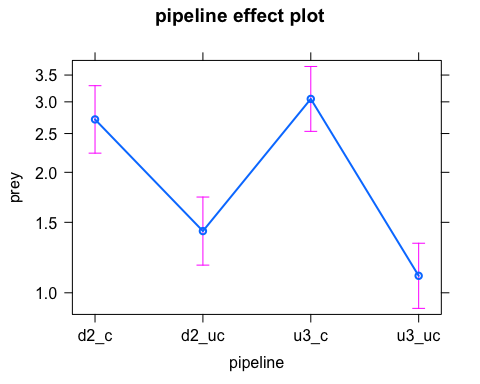


Unsurprisingly, cleaning increases the total proportion of prey ASVs in each sample. DADA2 has a higher proportion of prey ASVs than UNOISE3 for both clean and uncleaned datasets.

**Winner: DADA2 clean**

### A. Percent of reads that are prey

Again, we have been assuming that the total number of reads is independent of sequencing depth, which we know is not the case. Therefore, we ran the above reads model again correcting for sequencing depth (using an offset). These data can then be interpreted as the percent of all reads assigned to prey.


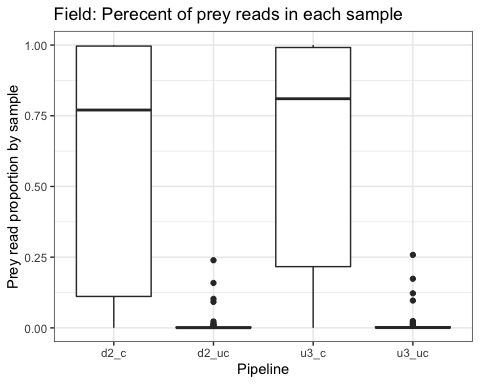


prey_rprop_mod <- glmmTMB(prey ~ pipeline + (1|sample),
 data = read_totals_samples,
 family = "genpois",
 offset = log(tot))

Cleaning increased the total proportion of reads that are assigned to prey ASVs. There is no difference in the proportion of prey reads in samples for either DADA2 or UNOISE3.

**Winner: cleaned datasets**

### H. Amount of known diet reads

We know we fed lab organisms a specific diet item (*Oxya japonica*), and so we should trust the pipeline that detects this prey item better.

Let’s look at detection across samples of this prey item (we subset our dataset to just be those that we fed the item to):


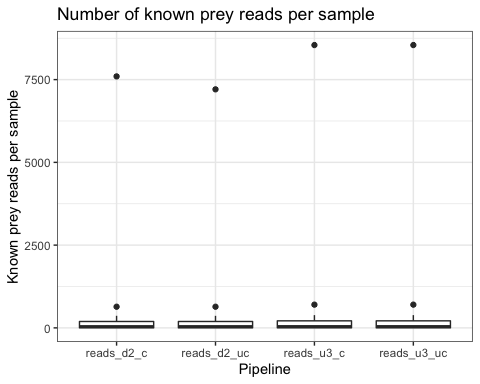


Again, we built a mixed model that we compared to a null model:

known_reads_mod <- glmmTMB(value ~ pipeline + (1|sample),
 data = known_reads_long,
 family = "genpois")

And looked at pairwise differences:

## contrast estimate SE df t.ratio p.value
## reads_d2_c - reads_d2_uc 0.040970 0.00610 70 6.712 <.0001
## reads_d2_c - reads_u3_c -0.111342 0.00588 70 -18.935 <.0001
## reads_d2_c - reads_u3_uc -0.111167 0.00588 70 -18.904 <.0001
## reads_d2_uc - reads_u3_c -0.152312 0.00595 70 -25.616 <.0001
## reads_d2_uc - reads_u3_uc -0.152137 0.00595 70 -25.586 <.0001
## reads_u3_c - reads_u3_uc 0.000175 0.00571 70 0.031 1.0000
##
## Results are given on the log (not the response) scale.
## P value adjustment: tukey method for comparing a family of 4 estimates


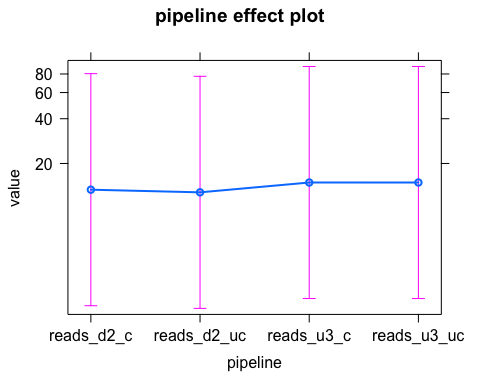


Cleaned datasets have significantly more reads of the known prey items per sample for DADA2, however cleaning did not increase detection of known prey for UNOISE3. UNOISE3 has better detection of known prey items.

## Pipeline Performance Summary

Compiling the results from this, we get a summary of all the measures of pipeline performance. In the case of a non-significant difference between pipelines, the pipeline with the most parsimonious protocol (i.e. no cleaning) will win. In the case that the parsimony is the same between pipelines, none is considered to be outperforming. (in the following table, an “X” designates the best performing pipeline, either by significant difference between groups or because it is the most parsimonious of a tie, “NS” signifies that these pipelines performed equally well to the winning, parsimonious pipeline, and “TIE” signifies that these pipelines performed equally well and have the same parsimony.)

| Measure/Pipeline | DADA2 UC | UNOISE3 UC | DADA2 C | UNOISE3 C |
| --- | --- | --- | --- | --- |
| Positive control ASVs |  | X |  |  |
| Negative conotrol ASVs | X |  | NS | NS |
| Total ASVs | X |  |  |  |
| ASVs per sample |  | X |  | NS |
| Prey ASVs |  | X |  |  |
| Prey reads |  | X |  |  |
| Prey ASV % |  |  | X |  |
| Prey read % |  |  | TIE | TIE |
| Known Diet |  | X |  | NS |
| NA | NA | NA | NA | NA |
| NA | NA | NA | NA | NA |

From this, we see that in most cases, the uncleaned pipelines perform equally well or better than the cleaned pipelines. So cleaning does not add much to our bioinformatic performance nor our ecological inference from this dataset. As a result, for our analyses of sterilized vs. unsterilized individuals (both field and lab), the best options in terms of time are both unclean DADA2 and UNOISE3. The “winner” between these two pipelines is unclear - UNOISE3 outperformed ecologically (providing more diet data to work with); conversely, DADA2 outperformed bioinformatically (controls mapped more accurately).

#### Takeaway: either UNOISE3 or DADA2 will give you good diet data with good specificity. The choice, then, may be dependent on access rather than performance. While DADA2 is open access and user-friendly in R, it takes longer to run than UNOISE3; conversely, while UNOISE3 runs much more quickly, it is only free in a 32-bit version that does not run on some new operating systems (e.g. 64-bit Mac Catalina OS). These and other costs-benefits are reviewed more thoroughly in Nearing et al. (2018)

**Literature:**

Bushnell, B. (2019). *BBMap*.

Callahan, B. J., McMurdie, P. J., Rosen, M. J., Han, A. W., Johnson, A. J. A., & Holmes, S. P. (2016). DADA2: High-resolution sample inference from Illumina amplicon data. *Nature Methods*, *13*(7), 581–583. https://doi.org/10.1038/nmeth.3869

Edgar, R. C. (2016). UNOISE2: improved error-correction for Illumina 16S and ITS amplicon sequencing. *BioRxiv*, 081257. https://doi.org/10.1101/081257

Hsieh, T. C., & Chao, A. (2017). Rarefaction and extrapolation: Making fair comparison of abundance-sensitive phylogenetic diversity among multiple assemblages. *Systematic Biology*, *66*(1), 100–111. https://doi.org/10.1093/sysbio/syw073

Nearing, J. T., Douglas, G. M., Comeau, A. M., & Langille, M. G. I. (2018). Denoising the Denoisers: An independent evaluation of microbiome sequence error- correction approaches. *PeerJ*, *2018*(8), 1–22. https://doi.org/10.7717/peerj.5364

# Appendix S2: BBSplit Methods

BBSplit maps sequences to reference databases of sequences provided by the user. The output of BBSplit is a file of sequences that have mapped to each reference database and a file for all sequences that do not map to any reference databases.

We decided to build our BBSplit reference databases based on sequences we already knew to be a part of our dataset (i.e. the output of a previous denoising pipeline). Because dada2 provides more ASVs than unoise3 (Nearing et al., 2018), we chose to use the dada2 ASV list to create our reference databases. We split the output of the MEGAN taxonomic alignment into sequences that mapped to the predator (*Heteropoda venatoria*) and those that mapped to prey (Supplement R code). We know that our predator *H. venatoria* is the only member of its genus and family on Palmyra Atoll, and so sequences that matched these higher classifications in MEGAN (Genus: *Heteropoda* or Family: Sparassidae; Handler et al.) were also split into the predator reference file. We then split our dada2 ASV list based on whether sequences mapped to these predator or prey ASV lists from MEGAN. Our final result was a set of two reference files, one including all predator ASVs, and one containing all prey ASVs.

After we had built these reference databases of predator and prey ASVs, we ran the BBSplit program using both the predator and prey reference files to map our trimmed sequences. We kept defaults for most settings of the BBSplit command, including that reads that ambiguously mapped to both databases should go in the best fit database (the default “ambiguous” and “ambiguous2” parameters equal to “best”). We kept this default because we expected that most sequences would be predator sequences. However, because raw sequences or sequences with high error rates had not been denoised yet, would not fit perfectly to the ASV list for predators, but would fit more closely to this ASV list than to the ASVs in the prey reference file (Code Supplement/Data). The output of BBSplit was one set of sequences that mapped to the prey reference ASVs, one that mapped to predator ASVs, and one that did not map to either of these (unmapped).

Although we were most interested in running dada2 and unoise3 again on the prey ASVs split with BBSplit, we also wanted to ensure that the splitting process did an accurate job of removing predator ASVS (i.e. predator sequence file should all map to predator after dada2 and unoise3), and that we weren’t missing any prey in the prey sequence file (by looking at the ASV list of the unmapped sequence file after dada2 and unoise3). Therefore, we ran dada2 and unoise3 against each mapped set of sequences: the prey-mapped sequences, the predator-mapped sequences, and the unmapped sequences. As a result, we had a total of six more ASV lists and ASV tables matched to each sample (3 from Dada2 and 3 from unoise3 in USEARCH). We then used BLAST and a database of all nucleotide sequences on GenBank (downloaded on November 20, 2019) and the BOLD IDEngine (accessed February 5-16, 2020) to match taxonomies to each of these ASV files. Again, we selected the subtree in MEGAN with likely prey items (Kingdom:Animalia, Clade: Bilateria) and exported the same files. For BOLD, we again used the Species Level Barcode Records database.

**Literature:**

Bushnell, B. (2019). *BBMap*.

Callahan, B. J., McMurdie, P. J., Rosen, M. J., Han, A. W., Johnson, A. J. A., & Holmes, S. P. (2016). DADA2: High-resolution sample inference from Illumina amplicon data. *Nature Methods*, *13*(7), 581–583. https://doi.org/10.1038/nmeth.3869

Edgar, R. C. (2016). UNOISE2: improved error-correction for Illumina 16S and ITS amplicon sequencing. *BioRxiv*, 81257. https://doi.org/10.1101/081257

Huson, D. H., Beier, S., Flade, I., Górska, A., El-Hadidi, M., Mitra, S., Ruscheweyh, H. J., & Tappu, R. (2016). MEGAN Community Edition - Interactive Exploration and Analysis of Large-Scale Microbiome Sequencing Data. *PLoS Computational Biology*, *12*(6), 1–12. https://doi.org/10.1371/journal.pcbi.1004957

Nearing, J. T., Douglas, G. M., Comeau, A. M., & Langille, M. G. I. (2018). Denoising the Denoisers: An independent evaluation of microbiome sequence error- correction approaches. *PeerJ*, *2018*(8), 1–22. https://doi.org/10.7717/peerj.5364

# Appendix S3: Model outputs for GLMMs

## 1. Prey Detection

### A. Mesocosm

For mesocosm consumers, the full model is of the form:

**presence ~ Sterilized, family = binomial**

where **presence** is the binary 0-1 detection of the offered prey item (*Oxya japonica*) in the sample and **Sterilized** is a two-level factor of either *surface sterilized* or *not surface sterilized*.

and the null model is:

**presence ~ 1, family = binomial**

####Model comparison with AICc

AICc(lab_detect_mod, lab_null_model)

## df AICc
## lab_detect_mod 2 22.54235
## lab_null_model 1 24.13599

#### Model summary

p-value of the surface sterilization fixed effect as marginally significant:

summary(lab_detect_mod)

## Family: binomial ( logit )
## Formula: presence ~ Sterilized
## Data: lab_detect
##
## AIC BIC logLik deviance df.resid
## 21.8 23.7 -8.9 17.8 17
##
##
## Conditional model:
## Estimate Std. Error z value Pr(>|z|)
## (Intercept) 2.303 1.049 2.195 0.0281 *
## SterilizedSS -2.303 1.265 -1.820 0.0687 .
## ---
## Signif. codes: 0 '***' 0.001 '**' 0.01 '*' 0.05 '.' 0.1 ' ' 1

#### Marginal means graph

A marginal means graph shows a decreased in detection with surface steriliation:

plot(allEffects(lab_detect_mod))


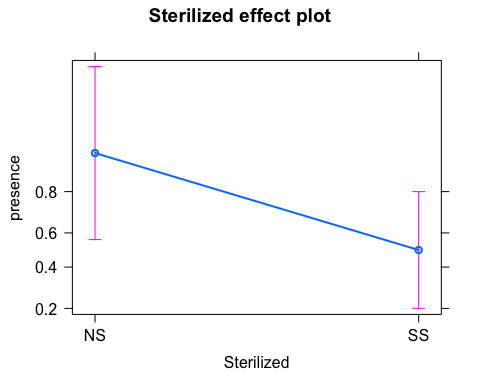


#### Model diagnostics

simulationOutput_lab <- simulateResiduals(fittedModel = lab_detect_mod)
fit_lab <- plot(simulationOutput_lab, asFactor=TRUE)


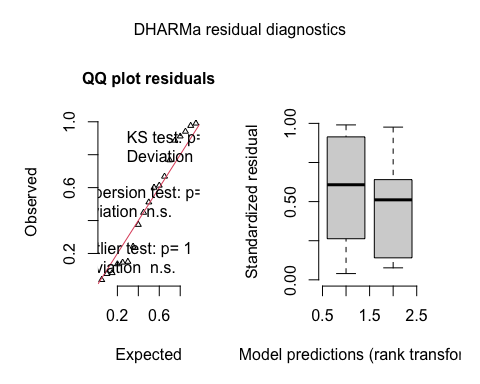


binned_residuals(lab_detect_mod)

## Ok: About 100% of the residuals are inside the error bounds.


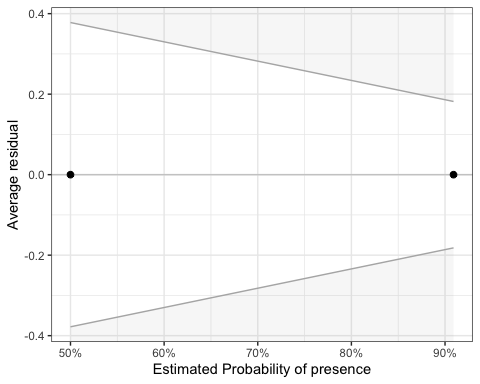


### B. Natural

For natural consumers, the full model is of the form:

**presence ~ Sterilized, family = binomial**

where **presence** is the binary 0-1 detection of any potential prey in the sample and **Sterilized** is a two-level factor of either *surface sterilized* or *not surface sterilized*.

and the null model is:

**presence ~ 1, family = binomial**

#### Model comparison with AICc

AICc(fld_detect_mod, fld_null_model)

## df AICc
## fld_detect_mod 2 29.69774
## fld_null_model 1 27.46236

#### Model summary

The sterilized term is non-significant:

summary(fld_detect_mod)

## Family: binomial ( logit )
## Formula: presence ~ Sterilized
## Data: field_detect
##
## AIC BIC logLik deviance df.resid
## 29.3 32.6 -12.7 25.3 35
##
##
## Conditional model:
## Estimate Std. Error z value Pr(>|z|)
## (Intercept) 2.14007 0.74755 2.863 0.0042 **
## SterilizedSS -0.06063 1.05893 -0.057 0.9543
## ---
## Signif. codes: 0 '***' 0.001 '**' 0.01 '*' 0.05 '.' 0.1 ' ' 1

summary(fld_null_model)

## Family: binomial ( logit )
## Formula: presence ~ 1
## Data: field_detect
##
## AIC BIC logLik deviance df.resid
## 27.3 29.0 -12.7 25.3 36
##
##
## Conditional model:
## Estimate Std. Error z value Pr(>|z|)
## (Intercept) 2.1102 0.5294 3.986 6.73e-05 ***
## ---
## Signif. codes: 0 '***' 0.001 '**' 0.01 '*' 0.05 '.' 0.1 ' ' 1

#### Model diagnostics

simulationOutput_fld <- simulateResiduals(fittedModel = fld_null_model)
fit_fld <- plot(simulationOutput_fld, asFactor=TRUE)


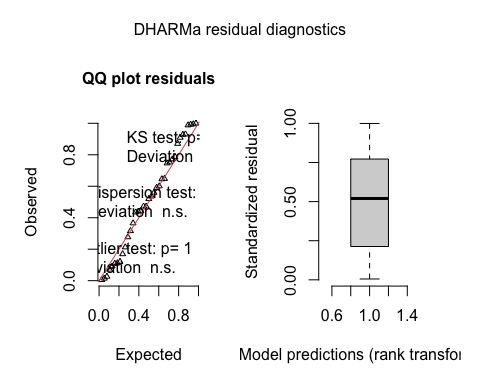


binned_residuals(fld_null_model)

## Ok: About 100% of the residuals are inside the error bounds.

## geom_path: Each group consists of only one observation. Do you need to adjust
## the group aesthetic?
## geom_path: Each group consists of only one observation. Do you need to adjust
## the group aesthetic?


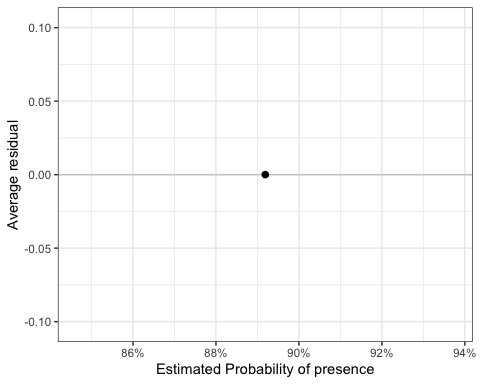


### C. Summary


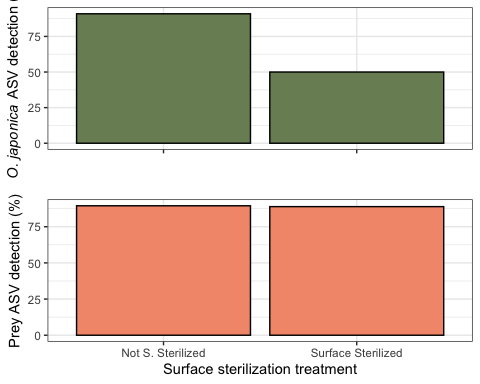


## 2. Prey DNA Abundance

### A. Mesocosm

For mesocosm consumers, the full model is of the form:

**offered prey ~ Sterilized, offset = log(total), family = “genpois”)**

where **offered prey** is the abundance of DNA from the offered prey item (*Oxya japonica*) in the sample, **Sterilized** is a two-level factor of either *surface sterilized* or *not surface sterilized*, and the offset term of **total** offset transforms the abundance value by the total DNA read abundance in the sample, since there is a huge spread in the raw DNA abundances.

and the null model is:

**offered prey ~ 1, offset = log(total), family = “genpois”)**

#### Model comparison with AICc

AICc(lab_mod, lab_null)

## df AICc
## lab_mod 3 180.2187
## lab_null 2 177.9251

#### Model summary

summary(lab_mod)

## Family: genpois ( log )
## Formula: known ~ Sterilized
## Data: lab_all_nz
## Offset: log(total)
##
## AIC BIC logLik deviance df.resid
## 177.8 179.7 -85.9 171.8 11
##
##
## Overdispersion parameter for genpois family (): 5.66e+03
##
## Conditional model:
## Estimate Std. Error z value Pr(>|z|)
## (Intercept) -4.6549 0.9358 -4.974 6.55e-07 ***
## SterilizedSS -0.4906 0.5111 -0.960 0.337
## ---
## Signif. codes: 0 '***' 0.001 '**' 0.01 '*' 0.05 '.' 0.1 ' ' 1

summary(lab_null)

## Family: genpois ( log )
## Formula: known ~ 1
## Data: lab_all_nz
## Offset: log(total)
##
## AIC BIC logLik deviance df.resid
## 176.8 178.1 -86.4 172.8 12
##
##
## Overdispersion parameter for genpois family (): 5.92e+03
##
## Conditional model:
## Estimate Std. Error z value Pr(>|z|)
## (Intercept) -4.7723 0.9512 -5.017 5.24e-07 ***
## ---
## Signif. codes: 0 '***' 0.001 '**' 0.01 '*' 0.05 '.' 0.1 ' ' 1

#### Model diagnostics

plot(residuals(lab_null))


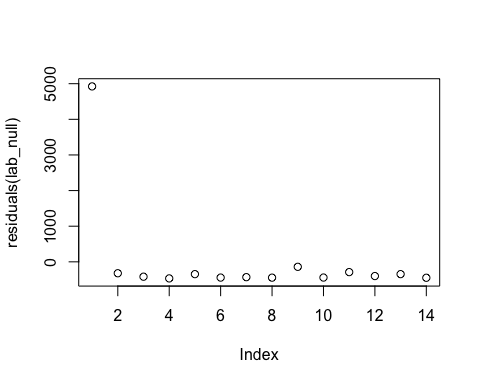


simulationOutput <- simulateResiduals(fittedModel = lab_null)
fit <- plot(simulationOutput, asFactor=TRUE)


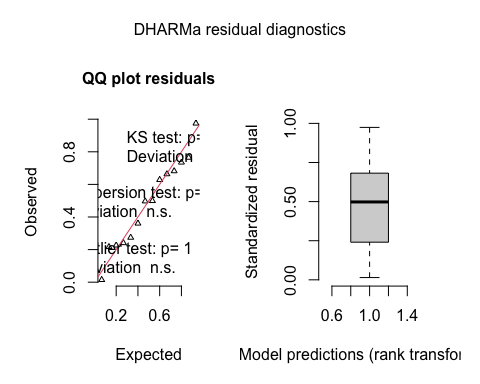


zi <- testZeroInflation(simulationOutput)


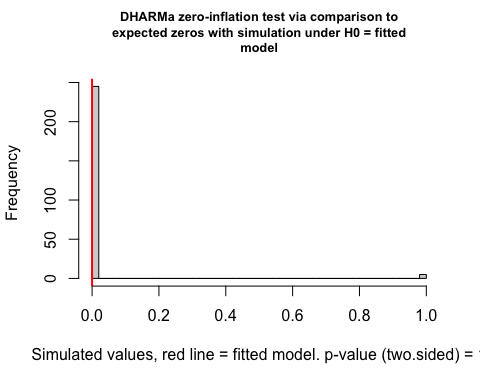


od <- testDispersion(simulationOutput)


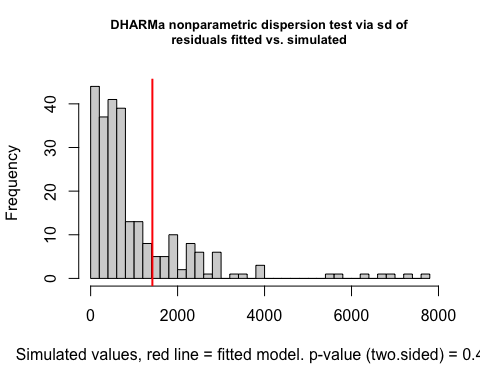


### B. Natural

For natural consumers, the full model is of the form:

**prey ~ Sterilized, offset = log(total), family = “genpois”)**

where **prey** is the abundance of potential prey DNA in the sample, **Sterilized** is a two-level factor of either *surface sterilized* or *not surface sterilized*, and the offset term of **total** offset transforms the abundance value by the total DNA read abundance in the sample, since there is a huge spread in the raw DNA abundances.

and the null model is:

**offered prey ~ 1, offset = log(total), family = “genpois”)**

#### Model comparison with AICc

AIC(fld_mod, fld_null)

## df AIC
## fld_mod 3 355.8463
## fld_null 2 353.8582

#### Model summary

summary(fld_mod)

## Family: genpois ( log )
## Formula: prey ~ Sterilized
## Data: fld_all_nz
## Offset: log(total)
##
## AIC BIC logLik deviance df.resid
## 355.8 360.3 -174.9 349.8 30
##
##
## Overdispersion parameter for genpois family (): 1.37e+04
##
## Conditional model:
## Estimate Std. Error z value Pr(>|z|)
## (Intercept) -3.8924 1.1468 -3.394 0.000688 ***
## SterilizedSS -0.0346 0.3178 -0.109 0.913303
## ---
## Signif. codes: 0 '***' 0.001 '**' 0.01 '*' 0.05 '.' 0.1 ' ' 1

summary(fld_null)

## Family: genpois ( log )
## Formula: prey ~ 1
## Data: fld_all_nz
## Offset: log(total)
##
## AIC BIC logLik deviance df.resid
## 353.9 356.9 -174.9 349.9 31
##
##
## Overdispersion parameter for genpois family (): 1.37e+04
##
## Conditional model:
## Estimate Std. Error z value Pr(>|z|)
## (Intercept) -3.909 1.137 -3.438 0.000586 ***
## ---
## Signif. codes: 0 '***' 0.001 '**' 0.01 '*' 0.05 '.' 0.1 ' ' 1

#### Model diagnostics

plot(residuals(fld_null))


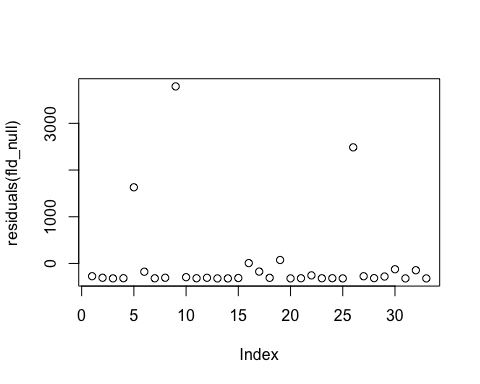


simulationOutput <- simulateResiduals(fittedModel = fld_null)
fit <- plot(simulationOutput, asFactor=TRUE)


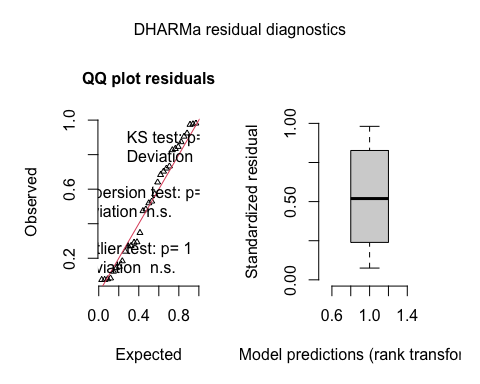


zi <- testZeroInflation(simulationOutput)


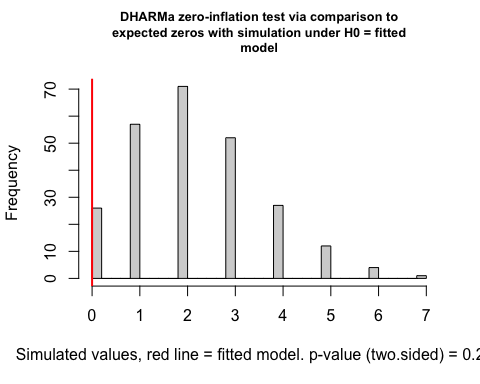


od <- testDispersion(simulationOutput)


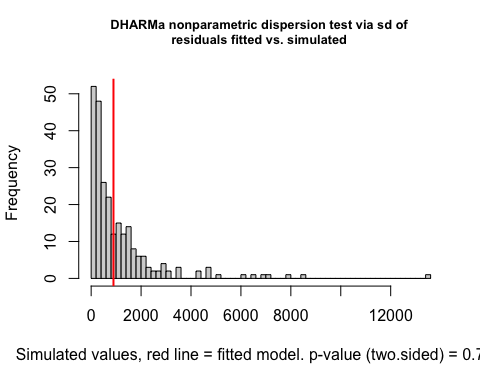


### C. Summary


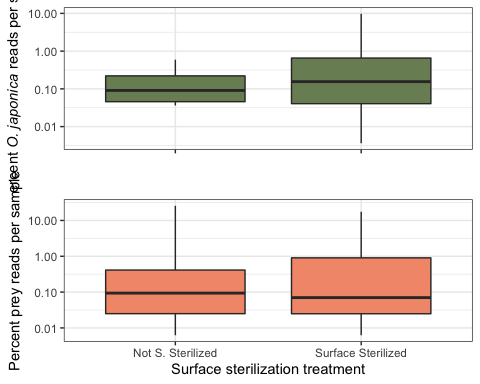


## 3. Natural: Prey DNA Richness and Composition

### A. Richness

We looked at richness of diet within each natural predator diet, with richness being the richness of family-level taxonomic assignments in each sample. The full model for richness is:

**SR ~ Sterilized, family = poisson**

where **SR** is taxonomic richness in a sample (concatenated at the family level) and **Sterilized** is a two-level factor of either *surface sterilized* or *not surface sterilized*.

and the null model is:

**SR ~ Sterilized, family = poisson**

#### Model comparison with AICc

AICc(rich_mod, rich_null)

## df AICc
## rich_mod 2 135.8017
## rich_null 1 133.5740

#### Model summary

#based on this summary, surface sterilizatoin treatment is
#non-significant.
summary(rich_mod)

## Family: poisson ( log )
## Formula: SR ~ Sterilized
## Data: richness
##
## AIC BIC logLik deviance df.resid
## 135.4 138.7 -65.7 131.4 35
##
##
## Conditional model:
## Estimate Std. Error z value Pr(>|z|)
## (Intercept) 0.74444 0.15811 4.708 2.5e-06 ***
## SterilizedSS -0.02389 0.22809 -0.105 0.917
## ---
## Signif. codes: 0 '***' 0.001 '**' 0.01 '*' 0.05 '.' 0.1 ' ' 1

summary(rich_null)

## Family: poisson ( log )
## Formula: SR ~ 1
## Data: richness
##
## AIC BIC logLik deviance df.resid
## 133.5 135.1 -65.7 131.5 36
##
##
## Conditional model:
## Estimate Std. Error z value Pr(>|z|)
## (Intercept) 0.7329 0.1140 6.431 1.27e-10 ***
## ---
## Signif. codes: 0 '***' 0.001 '**' 0.01 '*' 0.05 '.' 0.1 ' ' 1

#### Model diagnostics

plot(residuals(rich_null))


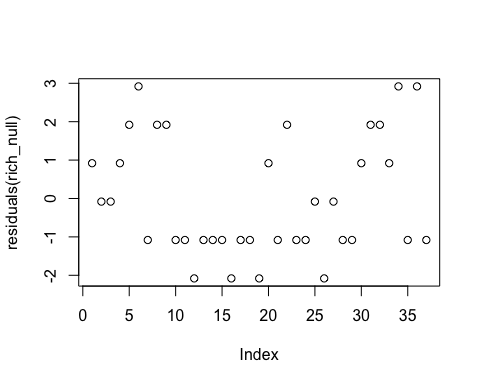


simulationOutput <- simulateResiduals(fittedModel = rich_null)
fit <- plot(simulationOutput, asFactor=TRUE)


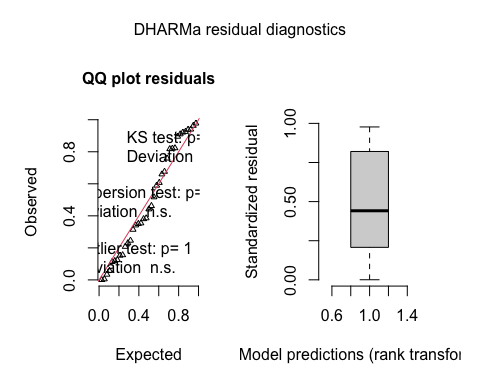


zi <- testZeroInflation(simulationOutput)


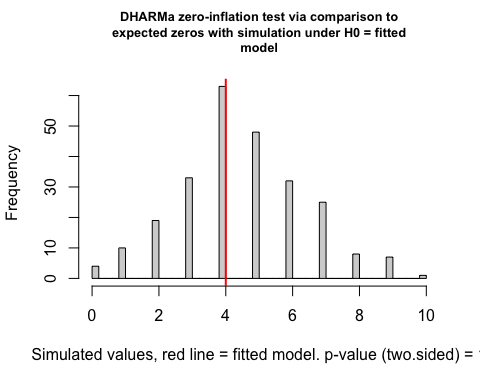


od <- testDispersion(simulationOutput)


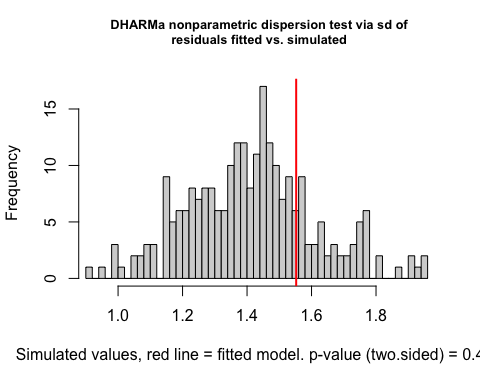


### B. Composition

We looked at presence-absence taxonomic composition of prey DNA using a GLMM-based PERMANOVA approach. Specifically, this GLMM is run by saying, how does the fixed effect of sterilization impact presence, with a random effects structure with both a random interept term for Family_ncbi (let each family have a different intercept) and a random slopes term for surface sterilization treatment (let each family’s relationship with with surface sterilization differ, ie let some families increase with surface sterilization, and others decrease)

The full model looks like:

**presence ~ Sterilized + (1+Sterilized|Family_ncbi), family = “binomial”**

And the null looks like:

**presence ~ 1 + (1|Family_ncbi), family = “binomial”**

#### Model comparison with AICc

AICc(comp_mod, comp_null)

## df AICc
## comp_mod 5 468.1095
## comp_null 2 462.0452

#### Model summary

summary(comp_null)

## Family: binomial ( logit )
## Formula: presence ~ 1 + (1 | Family_ncbi)
## Data: comp
##
## AIC BIC logLik deviance df.resid
## 462 471 -229 458 658
##
## Random effects:
##
## Conditional model:
## Groups Name Variance Std.Dev.
## Family_ncbi (Intercept) 0.5887 0.7673
## Number of obs: 660, groups: Family_ncbi, 20
##
## Conditional model:
## Estimate Std. Error z value Pr(>|z|)
## (Intercept) -2.2451 0.2282 -9.837 <2e-16 ***
## ---
## Signif. codes: 0 '***' 0.001 '**' 0.01 '*' 0.05 '.' 0.1 ' ' 1

summary(comp_mod) #sterilization term not significant

## Family: binomial ( logit )
## Formula: presence ~ Sterilized + (1 + Sterilized | Family_ncbi)
## Data: comp
##
## AIC BIC logLik deviance df.resid
## 468.0 490.5 -229.0 458.0 655
##
## Random effects:
##
## Conditional model:
## Groups Name Variance Std.Dev. Corr
## Family_ncbi (Intercept) 0.5985723 0.77367
## SterilizedSS 0.0001757 0.01326 -1.00
## Number of obs: 660, groups: Family_ncbi, 20
##
## Conditional model:
## Estimate Std. Error z value Pr(>|z|)
## (Intercept) -2.23856 0.27016 -8.286 <2e-16 ***
## SterilizedSS -0.01357 0.29549 -0.046 0.963
## ---
## Signif. codes: 0 '***' 0.001 '**' 0.01 '*' 0.05 '.' 0.1 ' ' 1

#### Model diagnostics

binned_residuals(comp_null)

## Warning: About 90% of the residuals are inside the error bounds (~95% or higher would be good).


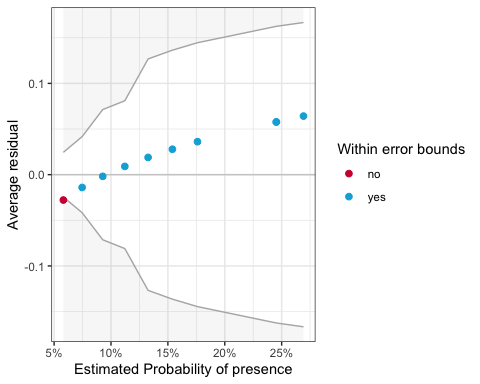


simulationOutput <- simulateResiduals(fittedModel = comp_null)
fit <- plot(simulationOutput, asFactor=TRUE)


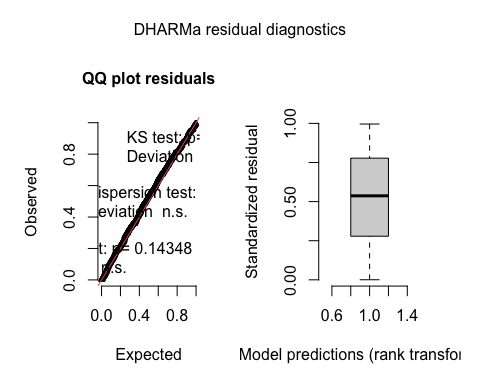


#### Supplementary: Compare to adonis()

The results are similar with an adonis() call from vegan, with the structure:

**comp1 ~ Sterilized, data = meta_field, dist = “jaccard”, binary = TRUE**

Where **comp1** is a matrix of interactions by individual, **Sterilized** is a binary fixed effect. We selected the Jaccard dissimilarlity index (indicating that data are presence-absence with binary=TRUE) since the Jaccard dissimilarity index is functionally Bray-Curtis dissimilarity, but better suited for presence-absence data

adonis(comp1 ~ Sterilized, data = meta_field, dist = "jaccard", binary = TRUE)

##
## Call:
## adonis(formula = comp1 ~ Sterilized, data = meta_field, dist = "jaccard", binary = TRUE)
##
## Permutation: free
## Number of permutations: 999
##
## Terms added sequentially (first to last)
##
## Df SumsOfSqs MeanSqs F.Model R2 Pr(>F)
## Sterilized 1 0.0462 0.04625 0.113 0.00363 0.999
## Residuals 31 12.6870 0.40926 0.99637
## Total 32 12.7333 1.00000

### C. Summary


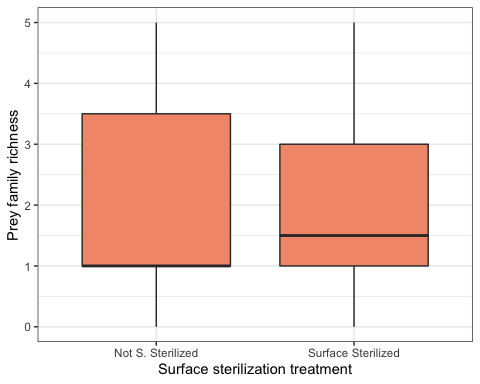


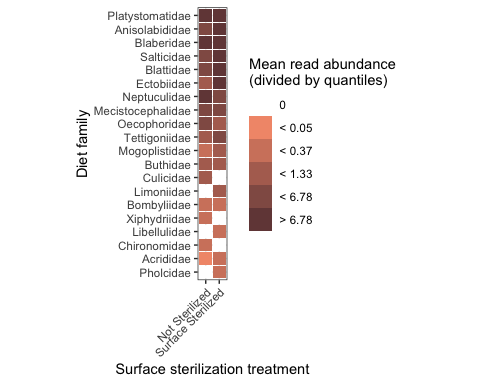


# Appendix S4: Model outputs for supplementary data analyses

## 1. DNA abundances

### A. Mesocosm all prey

For mesocosm consumers, we also examined whether all prey DNA abundance was altered by surface sterilization as well as examined whether surface sterilization altered the amount of non-diet (here, including fungi and likely endoparasites) DNA.

The all prey model comparisons included a full model: **prey ~ Sterilized, offset = log(total), family = “genpois”**

and a null model: **prey ~ 1, offset = log(total), family = “genpois”**

Where **prey** was all prey DNA abundance (including offered prey item, *Oxya japonica*), **Sterilized** is a binary factor including either *surface sterilized* or *not surface sterilized*, and the data are transformed from raw abundances (with wide ranges) by correcting with an offset of the total DNA abundance in each sample.

AICc(lab_mod, lab_null)

## df AICc
## lab_mod 3 180.2187
## lab_null 2 177.9251

summary(lab_all_mod)

## Family: genpois ( log )
## Formula: prey ~ Sterilized
## Data: lab_all_prey
## Offset: log(total)
##
## AIC BIC logLik deviance df.resid
## 241.6 244.3 -117.8 235.6 15
##
##
## Overdispersion parameter for genpois family (): 4.67e+03
##
## Conditional model:
## Estimate Std. Error z value Pr(>|z|)
## (Intercept) -4.6961 0.7592 -6.186 6.17e-10 ***
## SterilizedSS -0.2255 0.4005 -0.563 0.573
## ---
## Signif. codes: 0 '***' 0.001 '**' 0.01 '*' 0.05 '.' 0.1 ' ' 1

summary(lab_all_null)

## Family: genpois ( log )
## Formula: prey ~ 1
## Data: lab_all_prey
## Offset: log(total)
##
## AIC BIC logLik deviance df.resid
## 240.0 241.7 -118.0 236.0 16
##
##
## Overdispersion parameter for genpois family (): 4.68e+03
##
## Conditional model:
## Estimate Std. Error z value Pr(>|z|)
## (Intercept) -4.7779 0.7481 -6.387 1.7e-10 ***
## ---
## Signif. codes: 0 '***' 0.001 '**' 0.01 '*' 0.05 '.' 0.1 ' ' 1

plot(residuals(lab_all_null))


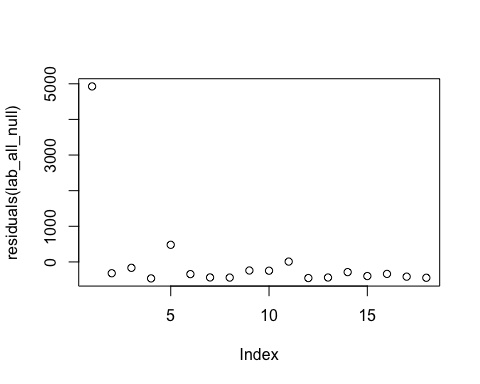


simulationOutput <- simulateResiduals(fittedModel = lab_all_null)
fit <- plot(simulationOutput, asFactor=TRUE)


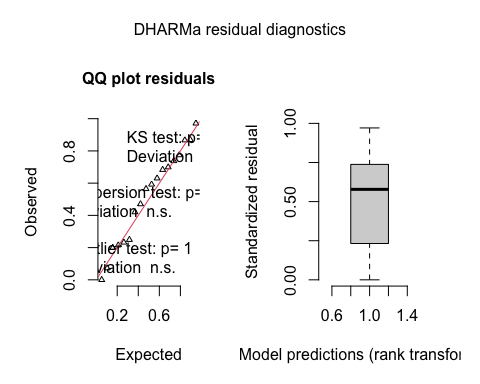


zi <- testZeroInflation(simulationOutput)


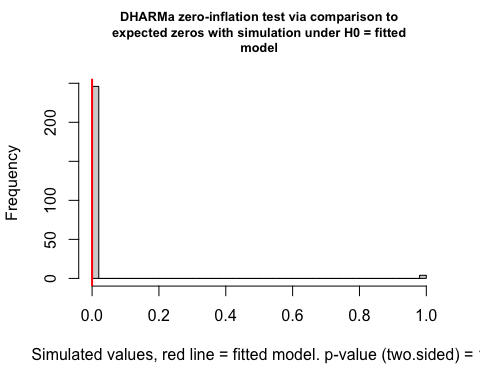


od <- testDispersion(simulationOutput)


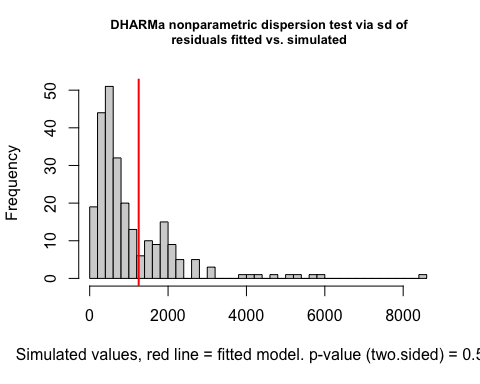


### B. Mesocosm non-diet

We examined non-diet DNA abundance in mesocosm consumers with a full-null model comparison.

The non-diet model comparisons included a full model: **nondiet ~ Sterilized, offset = log(total), family = “genpois”**

and a null model: **nondiet ~ 1, offset = log(total), family = “genpois”**

Where **nondiet** was all DNA abundance with taxonomic matches that were not diet items (here, fungi and likely endoparasites), **Sterilized** is a binary factor including either *surface sterilized* or *not surface sterilized*, and the data are transformed from raw abundances (with wide ranges) by correcting with an offset of the total DNA abundance in each sample.

AICc(lab_nd_mod, lab_nd_null)

## df AICc
## lab_nd_mod 3 241.5304
## lab_nd_null 2 240.0623

summary(lab_nd_mod)

## Family: genpois ( log )
## Formula: nondiet ~ Sterilized
## Data: lab_all
## Offset: log(total)
##
## AIC BIC logLik deviance df.resid
## 239.9 242.8 -117.0 233.9 16
##
##
## Overdispersion parameter for genpois family (): 472
##
## Conditional model:
## Estimate Std. Error z value Pr(>|z|)
## (Intercept) -5.5559 0.3859 -14.396 <2e-16 ***
## SterilizedSS -0.4104 0.3545 -1.158 0.247
## ---
## Signif. codes: 0 '***' 0.001 '**' 0.01 '*' 0.05 '.' 0.1 ' ' 1

summary(lab_nd_null)

## Family: genpois ( log )
## Formula: nondiet ~ 1
## Data: lab_all
## Offset: log(total)
##
## AIC BIC logLik deviance df.resid
## 239.3 241.2 -117.7 235.3 17
##
##
## Overdispersion parameter for genpois family (): 505
##
## Conditional model:
## Estimate Std. Error z value Pr(>|z|)
## (Intercept) -5.709 0.381 -14.98 <2e-16 ***
## ---
## Signif. codes: 0 '***' 0.001 '**' 0.01 '*' 0.05 '.' 0.1 ' ' 1

plot(residuals(lab_nd_null))


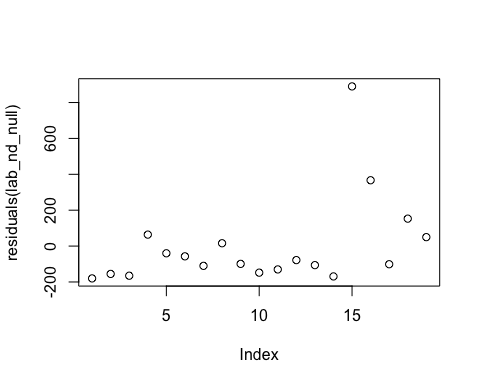


simulationOutput <- simulateResiduals(fittedModel = lab_nd_null)
fit <- plot(simulationOutput, asFactor=TRUE)


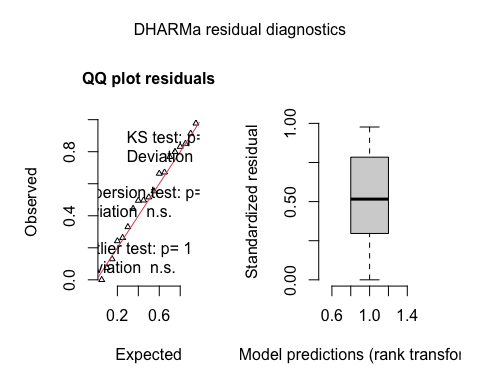


zi <- testZeroInflation(simulationOutput)


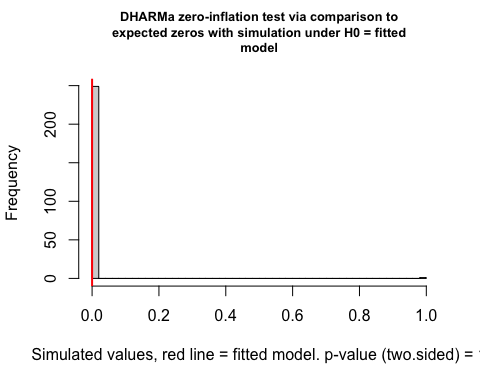


od <- testDispersion(simulationOutput)


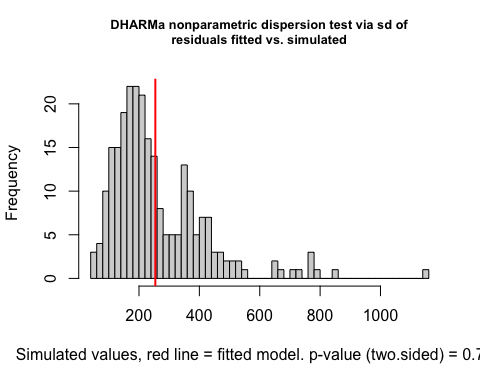


###C. Natural non-diet

We examined non-diet DNA abundance in natural environment consumers with a full-null model comparison.

The non-diet model comparisons included a full model: **nondiet ~ Sterilized, offset = log(total), family = “genpois”**

and a null model: **nondiet ~ 1, offset = log(total), family = “genpois”**

Where **nondiet** was all DNA abundance with taxonomic matches that were not diet items (here, fungi and likely endoparasites), **Sterilized** is a binary factor including either *surface sterilized* or *not surface sterilized*, and the data are transformed from raw abundances (with wide ranges) by correcting with an offset of the total DNA abundance in each sample.

AICc(fld_nd_mod, fld_nd_null)

## df AICc
## fld_nd_mod 3 296.0253
## fld_nd_null 2 294.6854

summary(fld_nd_mod)

## Family: genpois ( log )
## Formula: nondiet ~ Sterilized
## Data: fld_all
## Offset: log(total)
##
## AIC BIC logLik deviance df.resid
## 295.3 300.1 -144.6 289.3 34
##
##
## Overdispersion parameter for genpois family (): 889
##
## Conditional model:
## Estimate Std. Error z value Pr(>|z|)
## (Intercept) -5.6013 0.7044 -7.952 1.83e-15 ***
## SterilizedSS -0.3576 0.3545 -1.009 0.313
## ---
## Signif. codes: 0 '***' 0.001 '**' 0.01 '*' 0.05 '.' 0.1 ' ' 1

summary(fld_nd_null)

## Family: genpois ( log )
## Formula: nondiet ~ 1
## Data: fld_all
## Offset: log(total)
##
## AIC BIC logLik deviance df.resid
## 294.3 297.6 -145.2 290.3 35
##
##
## Overdispersion parameter for genpois family (): 902
##
## Conditional model:
## Estimate Std. Error z value Pr(>|z|)
## (Intercept) -5.7594 0.6951 -8.285 <2e-16 ***
## ---
## Signif. codes: 0 '***' 0.001 '**' 0.01 '*' 0.05 '.' 0.1 ' ' 1

plot(residuals(fld_nd_null))


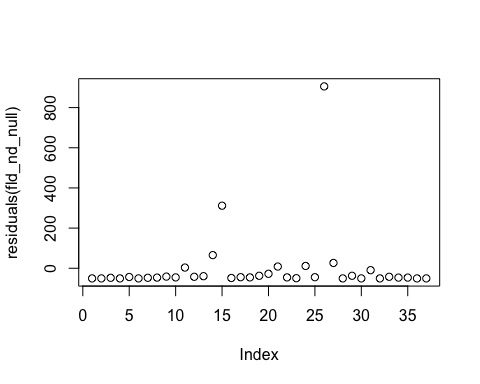


simulationOutput <- simulateResiduals(fittedModel = fld_nd_null)
fit <- plot(simulationOutput, asFactor=TRUE)


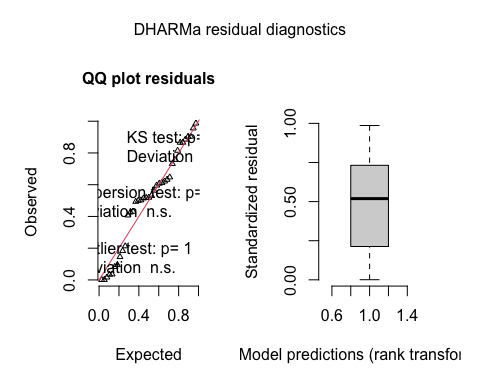


zi <- testZeroInflation(simulationOutput)


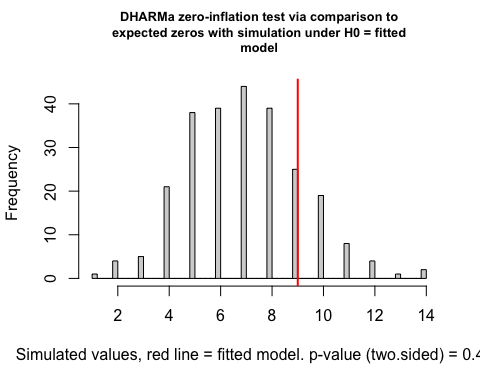


od <- testDispersion(simulationOutput)


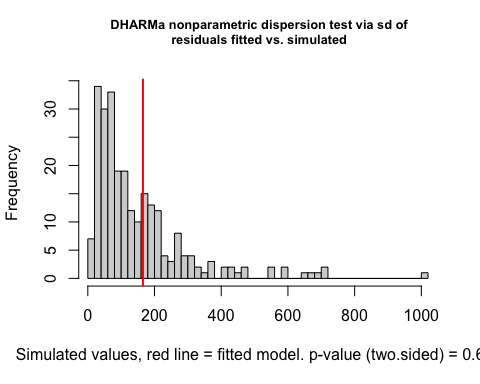


### D. Summary

## Warning: Transformation introduced infinite values in continuous y-axis

## Warning: Removed 9 rows containing non-finite values (stat_boxplot).


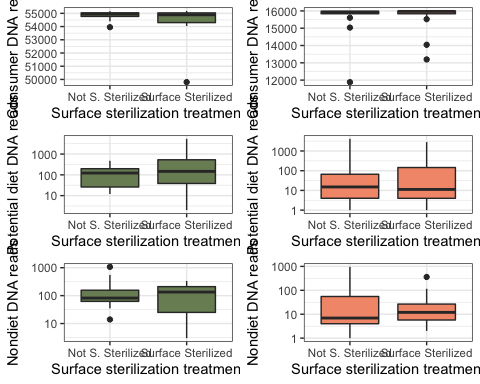


## 2. Natural abundance-based diet composition

We also performed an abundance-based PERMANOVA with a GLMM to see if abundance-based composition was influenced by surface sterilization. Because abundance can be due to many factors not related to diet importance (e.g. time since consumption, prey biomass, primer bias, random amplification and sequencing processes), this is not our primary analysis, but shows similar results.

Again, we ran a GLMM full model:

**reads ~ Sterilized + (1+Sterilized|Family_ncbi), family = “genpois”**

Where **reads** was the abundance of that diet item (again, concatenated at the family level taxonomic ID), **Sterilized** is a binary term of either *surface sterilized* or *not surface sterilized*. The random effects structure specifies that each family may have a distinct response to surface sterilization such that each family’s response can vary in magnitude (random intercept term of Family_ncbi) and that surface sterilization can either lead to increases or decreases in that family’s abundance (the random slope term of Sterilized).

and the null model:

**reads ~ 1 + (1|Family_ncbi), family = “genpois”**

AICc(bray_mod, bray_null)

## df AICc
## bray_mod 6 1107.984
## bray_null 3 1101.960

summary(bray_null)

## Family: genpois ( log )
## Formula: reads ~ 1 + (1 | Family_ncbi)
## Data: comp
##
## AIC BIC logLik deviance df.resid
## 1101.9 1115.4 -548.0 1095.9 657
##
## Random effects:
##
## Conditional model:
## Groups Name Variance Std.Dev.
## Family_ncbi (Intercept) 0.4922 0.7016
## Number of obs: 660, groups: Family_ncbi, 20
##
## Overdispersion parameter for genpois family (): 1.54e+04
##
## Conditional model:
## Estimate Std. Error z value Pr(>|z|)
## (Intercept) 2.529 1.219 2.075 0.038 *
## ---
## Signif. codes: 0 '***' 0.001 '**' 0.01 '*' 0.05 '.' 0.1 ' ' 1

summary(bray_mod) #sterilization term not significant

## Family: genpois ( log )
## Formula: reads ~ Sterilized + (1 + Sterilized | Family_ncbi)
## Data: comp
##
## AIC BIC logLik deviance df.resid
## 1107.9 1134.8 -547.9 1095.9 654
##
## Random effects:
##
## Conditional model:
## Groups Name Variance Std.Dev. Corr
## Family_ncbi (Intercept) 0.524656 0.72433
## SterilizedSS 0.002147 0.04633 -1.00
## Number of obs: 660, groups: Family_ncbi, 20
##
## Overdispersion parameter for genpois family (): 1.54e+04
##
## Conditional model:
## Estimate Std. Error z value Pr(>|z|)
## (Intercept) 2.5345 1.2261 2.067 0.0387 *
## SterilizedSS -0.0132 0.2816 -0.047 0.9626
## ---
## Signif. codes: 0 '***' 0.001 '**' 0.01 '*' 0.05 '.' 0.1 ' ' 1

plot(residuals(bray_null))


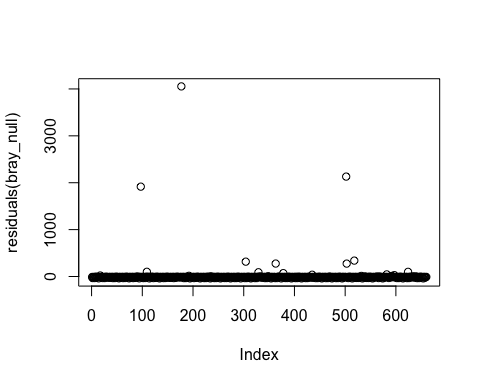


simulationOutput <- simulateResiduals(fittedModel = bray_null)
fit <- plot(simulationOutput, asFactor=TRUE)


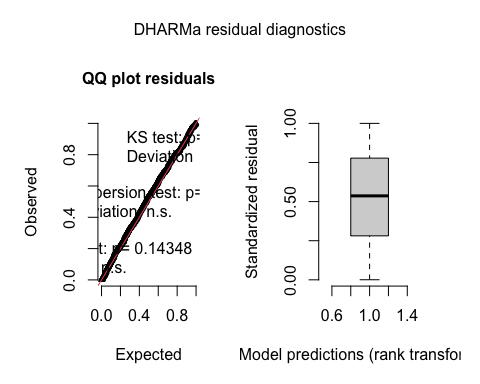


zi <- testZeroInflation(simulationOutput)


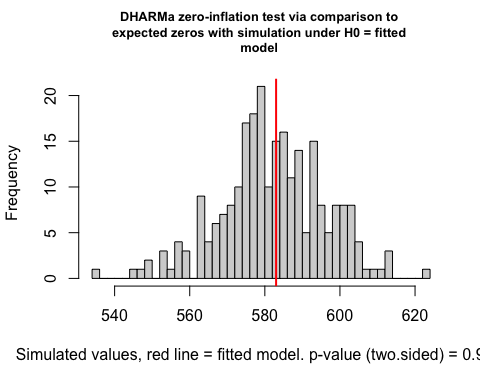


od <- testDispersion(simulationOutput)


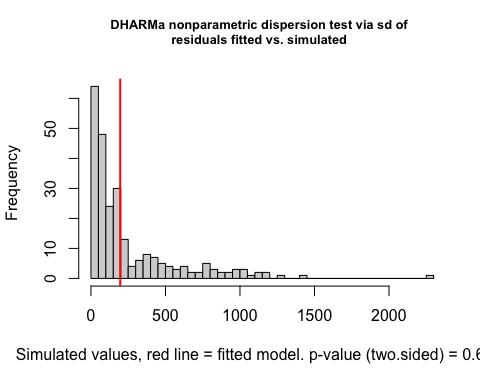


## 3. Mesocosm all prey composition

### A. Presence-based composition

For these analyses, we only performed analyses with adonis() in vegan, with a basic format of

**comp_lab ~ Sterilized, data = meta_lab, dist = “jaccard”, binary= TRUE**

with **comp_lab** being a matrix of diet presence by individual for all mesocosm consumers, **Sterilized** being a binary term indicating whether consumers were *surface sterilized* or *not surface sterilized*. We used a Jaccard dissimilarity metric, since this is an appropriate metric for presence-absence data (with binary=TRUE).

adonis(comp_lab ~ Sterilized, data = meta_lab, dist = "jaccard", binary = TRUE)

##
## Call:
## adonis(formula = comp_lab ~ Sterilized, data = meta_lab, dist = "jaccard", binary = TRUE)
##
## Permutation: free
## Number of permutations: 999
##
## Terms added sequentially (first to last)
##
## Df SumsOfSqs MeanSqs F.Model R2 Pr(>F)
## Sterilized 1 0.08728 0.087275 0.46217 0.02807 0.571
## Residuals 16 3.02143 0.188839 0.97193
## Total 17 3.10870 1.00000

### A. Abundance-based composition

We ran a similar adonis() on abundance data, this time with:

**abund_lab ~ Sterilized, data = meta_lab, dist = “bray”**

Where **abund_lab** is now a matrix of prey DNA abundances by sample and our dissimilarity metric is now Bray-Curtis, which is appropriate for abundance data.

adonis(abund_lab ~ Sterilized, data = meta_lab, dist = "bray")

##
## Call:
## adonis(formula = abund_lab ~ Sterilized, data = meta_lab, dist = "bray")
##
## Permutation: free
## Number of permutations: 999
##
## Terms added sequentially (first to last)
##
## Df SumsOfSqs MeanSqs F.Model R2 Pr(>F)
## Sterilized 1 0.6450 0.64499 1.8952 0.10591 0.062 .
## Residuals 16 5.4452 0.34032 0.89409
## Total 17 6.0902 1.00000
## ---
## Signif. codes: 0 '***' 0.001 '**' 0.01 '*' 0.05 '.' 0.1 ' ' 1

While this shows a marginally significant result, I’m not sure I trust this given that there are so few families in the mesocosm consumer diet contents:

lab %>%
 distinct(Family_ncbi) %>%
 tally(name = "Number of Families")

## Number of Families
## 1 7

And many of the families occur in one or a only a few individuals for the most part

lab %>%
 group_by(Family_ncbi) %>%
 summarise(Frequency = sum(reads >0))

## `summarise()` ungrouping output (override with `.groups` argument)

## # A tibble: 7 x 2
## Family_ncbi Frequency
## <chr> <int>
## 1 Acrididae 14
## 2 Anisolabididae 1
## 3 Blaberidae 8
## 4 Blattidae 1
## 5 Elateridae 1
## 6 Gekkonidae 1
## 7 Platystomatidae 2


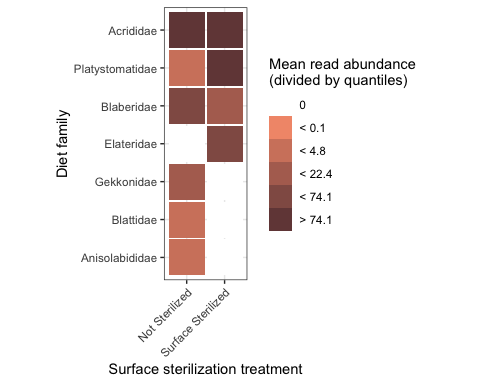


# Appendix S5: Supplementary Figures

Supplemental figures on DNA extraction and amplification protocols, as well as additional figures of diet composition and diversity for mesocosm and natural-environment consumers.

### 1. DNA cleaning with Ampure XP beads


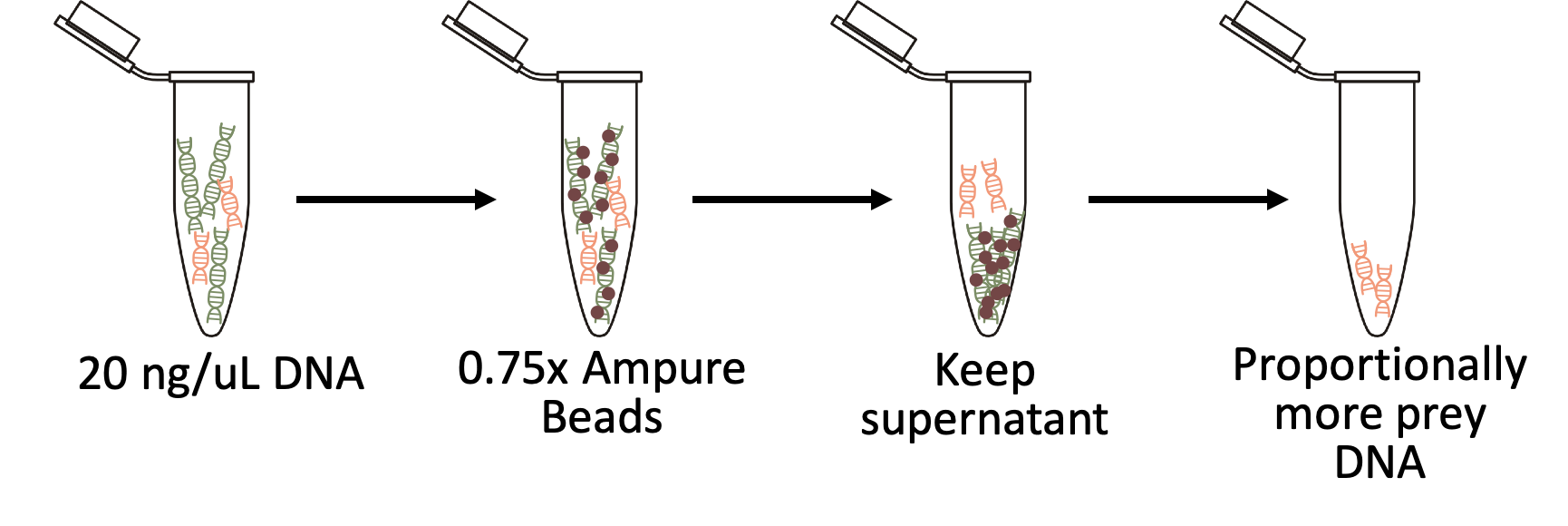


Supplementary Figure S1: Ampure XP bead cleaning of DNA to remove consumer DNA, motivated by results from Krehenwinkel et al. 2016.

### 2. Library preparation schematic

Supplementary Figure S2: Library prep, starting with attaching the CO1 primer pair with Illumina tag to diluted, bead-cleaned DNA. Then, this PCR product is bead cleaned at a 0.8x ratio and run through a subsequent PCR step to attach Illumina tag, index, and P5/P7 identifiers. This PCR product is then cleaned again at a 0.7x bead ratio, diluted to 5nM, and pooled for sequencing on an Illumina MiSeq.

### 3. Denoising algorithm process


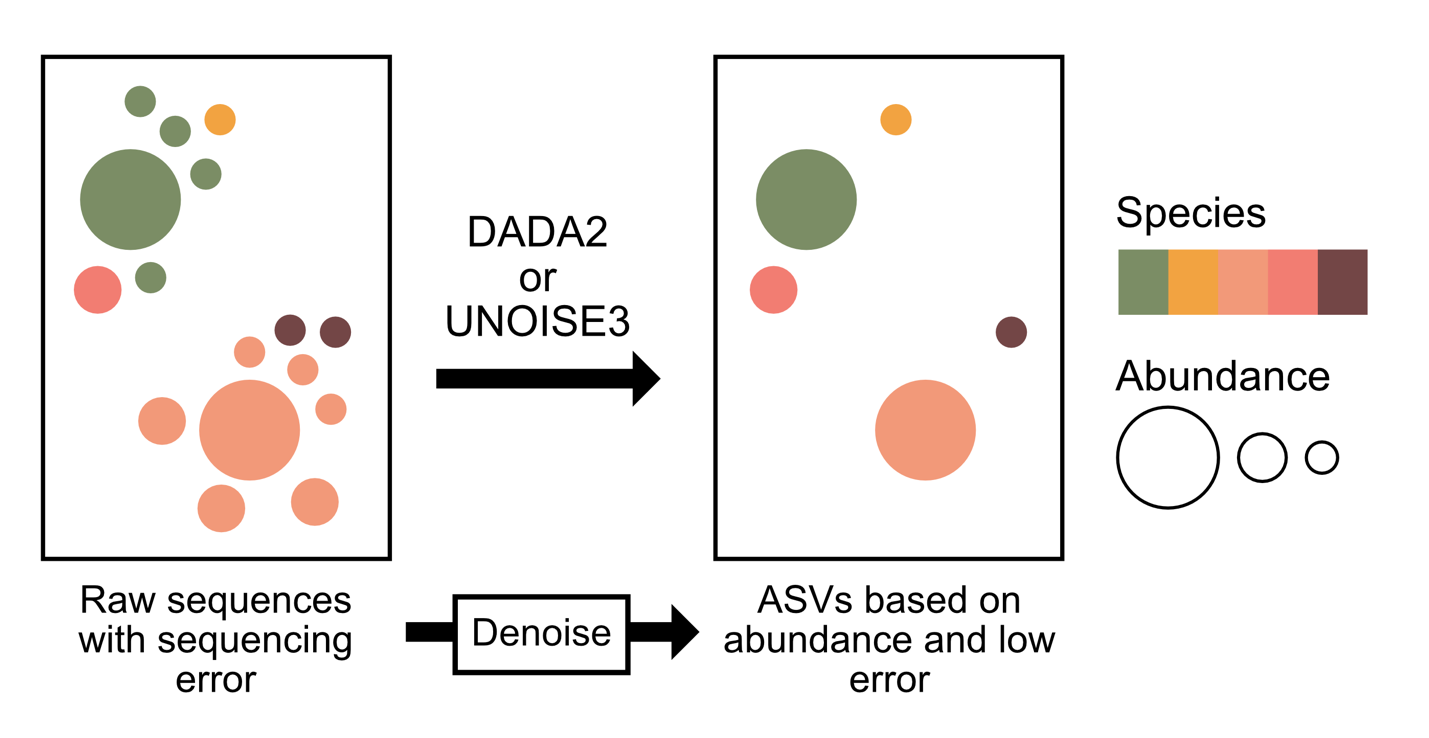


Supplementary Figure S3: Denoising algorithms like UNOISE3 and DADA2 take into account DNA sequence abundance and error rates to assign groups of similar sequences to one amplicon sequence variant (ASV). In this process, reads with sequencing and PCR point error are identified and removed.

### 4. Sequencing depth

Supplementary Figure S4: Sequencing depth of a) mesocosm and b) natural environment consumers determined via interpolation and extrapolation in the iNEXT package in R. All samples were sequenced to 99-100% sequencing depth. Each colored line corresponds to a consumer individual and the dashed vertical line represents the sequencing depth to which all samples were rarefied prior to analyses for each set of consumers.

### 5. All DNA source abundances

Supplementary Figure S5: Consumer DNA read abundances from A) mesocosm consumers and B) natural-environment consumers, potential diet DNA reads from C) mesocosm consumers and D) natural-environment consumers, and non-diet DNA read abundance for E) mesocosm consumers and F) natural-environment consumers that were and were not surface sterilized. The surface sterilized/not surface sterilized treatment groups are not significantly different for any type of other DNA.

### 6. Mesocosm consumer presence and abundance effect sizes

Supplementary Figure S6: The composition by-family of other diet in the mesocosm consumers, demonstrating that both presence- and abundance-based diet communities did not shift with surface sterilization treatment. (more positive means more present/abundant in unsterilized; more negative means more present/abundant in sterilized). Families are ranked by their overall presence in the population (A) or their overall abundance in the population (B) to demonstrate that there is no skew for relatively abundant or rare families.

### 7. Natural consumer presence and abundance effect sizes

Supplementary Figure S7: The composition by-family of diet in the natural environment consumers, demonstrating that both presence- and abundance-based diet communities did not shift with surface sterilization treatment. More positive values mean more present/abundant in non-sterilized; more negative values mean more present/abundant in surface sterilized consumers). Families are ranked by their overall presence in the population (A) or their overall abundance in the population (B) to demonstrate that there is no skew for relatively abundant or rare families.

### 8. Natural consumer presence and abundance of prey items by individual

Supplementary Figure S8: For natural environment consumers, per sample presence, abundance, and total richness of each diet family did not change with surface sterilization treatment.

### 9. Natural consumer presence and abundance of prey ASVs
