# Supplementary material for: Effects of consumer surface sterilization on diet DNA metabarcoding data of terrestrial invertebrates in natural environments and feeding trials
Source: Ecol Evol. 2021 Aug 9;11(17):12025–34. doi: 10.1002/ece3.7968 (PMC8427582; doi:10.1002/ece3.7968)
Supplement: Supplementary file 2 — Appendix S6 [file ECE3-11-12025-s001.docx]

**DNA Extraction and PCR Protocol (from raw tissue to PCR2 submission)**

***Instructions are organized into steps A – J, which include DNA extraction, quantifying, diluting, cleaning, and PCR steps.**

**A. Oono Lab CTAB Protocol* (Updated 5/2017 -Austen Apigo)**

***This protocol has modifications from the original protocol:**

**http://1000.fungalgenomes.org/home/protocols/high-quality-genomic-dna-extraction**

**-- see Sara Branco’s “Short CTAB Protocol”**

**For extractions, you will need:**

1. Water bath/Heat block
2. Fume hood
3. Pipettes and tips
4. Reagents (see below)
5. Tissue samples
6. Sterile 1.5 uL centrifuge tubes
7. Centrifuge
8. Orbital shaker
9. Vortex

**Extraction preparation:**

1. Set water bath to 65 C
2. Label two tubes per sample (1 with tissue, 1 empty for step #7)
3. Put 70% and 100% ethanol in -20C freezer
4. Make CTAB extraction buffer (for 100 ml)
   1. 2g CTAB powder (final concentration = 2% w/v)
   2. 28 ml 5M NaCl (final concentration = 1.4 M) 14 ml
   3. 4 ml 0.5M EDTA (final concentration = 20 mM) 2 ml
   4. 10 ml 1M Tris-HCl (pH 8; final concentration = 100 mM) 5 ml
   5. 58 ml Nuclease free water 29 ml
5. Working Buffer
   1. Add CTAB extraction buffer (500 uL/reaction) to 50 ml falcon tube
      1. Remember: there are two tubes of ground tissue per sample so --
         1. # of reactions = # of samples * 2
   2. Add PVP-40 (.02g per reaction)
   3. Put in water bath to dissolve (Use the thermometer attached to the water bath to hold falcon tube up vertically against the corner of water bath. Do NOT use the foam flotation pads or let the tube sit horizontally in the bath, the solution will leak out into water bath.)
   4. **AFTER WATER BATH:** Add 2.5 uL/reaction beta-mercaptoethanol to CTAB-PVP mix after PVP has dissolved
      1. It is better to add this last after the PVP is melted in the fume hood because the 2-mercapto is very volatile (i.e., smelly) and a hazardous chemical.

| Working Buffer | 10 samples | 15 samples | 20 samples | 25 samples | 45 samples | 50 samples | 100 samples |
| --- | --- | --- | --- | --- | --- | --- | --- |
| CTAB (ul) | 5,000 | 7,500 | 10,000 | 12,500 | 22,500 | 25,000 | 50,000 |
| PVP-40 (g) | 0.2 | 0.3 | 0.4 | 0.5 | 0.9 | 1 | 2 |
| B-mercaptoethanol (ul) | 25 | 38 | 50 | 63 | 113 | 125 | 250 |

**Extraction protocol:**

(All centrifugation steps are done at max speed - 12700 rpm)

1. **50-80 mg of tissue (ideally targeting hindgut region, and minimizing legs)** to each 1.5 ml microcentrifuge tube
2. Add **500 ul CTAB/PVP-40/B-mercaptoethanol solution**
3. Incubate at **65 C for 1 hour:**
   1. after 30 minutes, remove from water bath and grind with pestel – vortex
   2. after last 30 minutes, vortex again
   3. Double check caps are closed before vortexing, they may open due to heat
4. Make solution of 1:24 isoamyl alcohol-chloroform if you haven’t already done so.
5. Add **500 ul of 1:24 isoamyl-chloroform**
6. Place samples on orbital shaker for **20 minutes @ 200 rpm** (move orbital shaker to fume hood)
   1. Take off the flask rack and tape the tubes down horizontally.
   2. While samples are on the shaker, start aliquoting steps #9 and #10 to the third empty, labeled tube for each sample.
7. Spin down for **10 minutes** (microcentrifuge hinge up)
   1. If you put the microcentrifuge up when you centrifuge, the top phase will slant in such a way that it will be easier to get the supernatant.
8. Transfer **300 ul** of supernatant per tube to the second labeled tube
   1. Be sure to take only the top phase by keeping your pipet tip at the top of the liquid and working it down slowly as you pull up liquid in your pipet
9. Add **15 ul of 5M potassium acetate**
10. Add **200 ul isopropanol** and mix well (invert tube repeatedly)
11. Cold incubation in for **1 hour in -20C freezer**
12. Spin down **20 minutes** at max speed (microcentrifuge hinge down)
13. Discard **250 ul** of supernatant
14. Wash pellet with **250 ul of ice cold 70% ethanol** (mix by pipetting to dislodge pellet)
15. Spin down **3 minutes**
16. Discard **450 ul** supernatant
17. Wash pellet with **250 ul of ice cold 100% ethanol** (mix by pipetting to dislodge pellet)
18. Spin **2 minutes**
19. Discard all supernatant - use 20 ul pipet to remove all liquid
20. Let pellet dry in laminar flow hood for **30 minutes**
21. Reconstitute in **20 ul of TE buffer** (let pellet sit in TE overnight in the fridge and mix well by pipetting the next day before Qubit fluorometry)

**B. DNA Quantification and Dilution**

**For quantification, you will need:**

- - - 1. Qubit Fluorometer and qubit quantification kit for High Sensitivity DNA
      2. DNA stock solutions (from CTAB or DNA extraction kit in Step 1).
      3. TE buffer for dilution
      4. Falcon tube (for stock solution)
      5. Pipettes and tips

**Quantification protocol:**

Use the Qubit DNA setting with 1uL of sample per tube.

Make a Qubit working solution of Qubit reagents (1uL fluorescent: 99 uL of buffer). Make a few reactions more than you need (3-5).

Label Qubit tubes by sample and 2 for the standards.

Make standards by mixing 10uL of standard 1 and 190 uL of working solution, and 10uL of standard 2 and 190uL of working solution. Make sure to keep standard stocks in the fridge when not in use, and to shake both thoroughly before use.

Add 199uL of working solution to each of remaining Qubit tube

Mix each DNA sample well via pipette, and add 1uL of each DNA stock to each of the Qubit tubes.

Vortex each sample for 10 seconds on high and make sure that no bubbles remain in the bottom of tube.

Quantify DNA using Qubit fluorometer. First, read standards, and then read the remaining samples.

**Preparation for DNA Bead cleaning**

If consumer and prey are not taxonomically distinct, perform an AmPure XP bead clean up on 40uL diluted to 20ng/ul. Ampure beads preferentially bind to high molecular weight DNA strands – by cleaning with beads at this step, taking the supernatant from the process, proportionally more low molecular weight DNA (that of degraded prey tissue) is ideally in the sample (Krehenwinkel et al. 2017).

**C. Clean Raw DNA with AMPure XP beads**

**For Ampure cleaning, you will need:**

- - - 1. Ampure XP beads
      2. Magnetic rack
      3. Sterile PCR strips
      4. Reagents (see below)
      5. Centrifuge
      6. Sterile 1.5mL centrifuge tubes

**Ampure preparations:**

1. Wipe down pipettes and post PCR area with 70% ethanol and/or 10% bleach
2. Get 20ng/ul DNA from fridge and magnet plate from shelf
3. Aliquot out Ampure beads to a 1.5 mL tube.
4. Make fresh 80% ethanol
   1. I find it easiest to open many 1.5 mL tubes, then add 800 ul of 100% ethanol and 200 ul of NF water.
5. Make 10mM Tris (pH 8.5) Resuspension Buffer
6. Gently shake AmPure beads to resuspend (stored in fridge).

**Raw DNA Ampure protocol:**

1. Place each sample in a well in a UV-treated PCR strip, measuring how much of each sample you have.
2. Add Ampure beads to the tube in a **0.75uL beads: 1uL DNA** ratio. (using the measured amount you calculated). and mix by pipetting so solution is homogeneously brown
3. Let sit 5 minutes at room temperature.
4. Place on magnet plate. Let sit 2 minutes. Solution should become clear.
5. Pipette ~(volume – 5uL) into a new 1.5 tube.
6. Add **15 ul of 5M potassium acetate**
7. Add **200 ul isopropanol** and mix well (invert tube repeatedly)
8. Cold incubation in for **1 hour in -20C freezer**
9. Spin down **20 minutes** at max speed (microcentrifuge hinge down)
10. Discard **250 ul** of supernatant
11. Wash pellet with **250 ul of ice cold 70% ethanol** (mix by pipetting to dislodge pellet)
12. Spin down **3 minutes**
13. Discard **450 ul** supernatant
14. Wash pellet with **250 ul of ice cold 100% ethanol** (mix by pipetting to dislodge pellet)
15. Spin **2 minutes**
16. Discard all supernatant - use 20 ul pipet to remove all liquid
17. Let pellet dry in laminar flow hood for **30 minutes**
18. Reconstitute in **20 ul of TE buffer** (let pellet sit in TE overnight in the fridge and mix well by pipetting the next day before Qubit fluorometry)
19. **Cleaned DNA Quantification and Dilution:**
    - - 1. Quantify DNA using Qubit fluorometer with 1uL of sample per tube (following protocol in part B).
        2. Dilute 20uL to 10ngDNA/uL
        3. Put in freezer if not going to process soon -as DNA degrades over time. Otherwise -4C fridge is fine
20. **PCR1**

For both PCR steps, all primers should be diluted from the stock primer (which is at 100mM) to 10mM (10uL primer:90uL NF water).

(*note*: The first reaction contains primers that bind to a desired region in addition to a transposase tag. Conduct PCR reaction in duplicate for each sample. All precautions must be taken to prevent contamination, so work in UV Box.)

PCR1 Primers sequences (transposase in **bold**):

Metazoan (all animals) primers: mlCOIintF/Fol-degen-rev

PCR1-Fol-degen-rev **GTCTCGTGGGCTCGGAGATGTGTATAAGAGACAG**TANACYTCNGGRTGNCCRAARAAYCA

PCR1-mlCOIintF **TCGTCGGCAGCGTCAGATGTGTATAAGAGACAG**GGWACWGGWTGAACWGTWTAYCCYCC

**PCR1 Preparation:**

1. Gather the following in UV box:
   1. Filter tips
   2. Multichannel tips
   3. PCR strips and caps
   4. 1.5mL tubes for master mix - (I like to use the colored ones. The clear ones are hard to see in the ice.)
   5. Pipettes
   6. Multi-channel pipet
   7. Gloves
   8. Nuclease Free water
   9. GoTaq Green Master Mix
   10. Sharpie
   11. Tape
   12. Ice (can be found in soil room)
   13. Cold rack
2. Hang gloves a couple inches from the UV light using tape.
3. UV treat for 15-20 minutes. Turn off UV light before beginning any work.
4. Label PCR tubes/rack.
5. Make Master Mix (MM). Remove bottom section from the box and keep shield down to minimize contamination. Grab cold rack. Keep all reagents on ice.

NF water 9uL

GoTaq Green MM 12.5uL

PCR1-ITS1F-KYO1 1.25uL

PCR1-ITS2-KYO1 1.25uL

DNA (10ng) 1 uL (DNA conc. 10ng/uL)

**PCR1 Protocol:**

1. Put PCR tubes on cold rack. Add 24uL of MM to all tubes.
2. Add 1uL of DNA in duplicate to tubes.
3. Mix samples by pipetting with multichannel.
4. Spin down.
5. Run PCR Program AmtK_PCR1_46C program

95C 3:00

Repeat 35 times 95C :30

46C :30

72C 1:00

72C 5:00

4C forever

1. **CLEAN PCR 1 with Ampure Beads**

Add Ampure beads to the tube (**0.8 uL beads : 1uL DNA**) and mix by pipetting so solution is homogeneously brown (from Jen Smith 16S protocol)

Let sit 5 minutes at room temperature.

Place on magnet plate. Let sit 2 minutes. Solution should become clear.

Remove ~40 uL of solution so you do not disturb beads.

Add 100 uL of 80% ethanol and let sit 30s.

Remove all ethanol.

Repeat 6 and 7. Remove all ethanol.

Let samples sit 10 minutes in laminar flow hood to allow any residual ethanol to evaporate.

Take tubes off of magnet plate and add 20 uL of Resuspension Buffer (10 mM Tris). Mix by pipetting and let sit 2 minutes.

Let the TRIS sit for a few minutes if the dried beads are hard to re-suspend. Do NOT scrape the sides of the tubes to resuspend dried beads.

Put tubes back on magnet plate and let sit 1 minute.

Transfer elutant to new UV-treated PCR strip

***PCR products will degrade over time in the 4C fridge. Move to freezer if not planning to use.

1. **PCR2**

**PCR2 Preparation:**

Assign unique forward and reverse primer pairs (Illumina Nextera XT Index Kit v2 <https://support.illumina.com/content/dam/illumina-support/documents/documentation/chemistry_documentation/experiment-design/illumina-adapter-sequences-1000000002694-11.pdf>) to each sample for identification.

1. Work in UV Box.
2. Gather DNAs and reagents
3. Make Master Mix (MM)
   1. NF water 5uL
   2. GoTaq Green MM 12.5uL
   3. South Primer 1.25uL
   4. North Primer Add separately

**PCR2 Protocol:**

1. Add 18.75uL of MM to tubes
2. Add 1.25uL south primer to each tube
3. Add 5uL of PCR1 product
4. Mix samples by pipetting with multichannel.
5. Spin down
6. Run NGPCR2 Program then save in fridge until bead clean-up and quantification

95C 3:00

Repeat 10 times 95C :30

55C :30

72C :30

72C 5:00

4C forever

***PCR products will degrade over time in the 4C fridge. Move to freezer if not planning to use soon.

1. **Gel electrophoresis**

To verify that PCR2 successfully amplified, run samples through gel electrophoreses. Only keep samples for submission in which both duplicates successfully amplified. These will be combined during the final bead cleaning step.

For a standard large gel rig:

- - - 1. Make a 1.25% agarose gel by combining 300mL of 1x TAE Buffer with 3.75g of agarose powder (Bio-Rad Cat. #1613102). Microwave to combine.
      2. Add 9uL of gel red (3uL per 100mL TAE) and mix. Pour into gel rig and let solidify (~20-30 minutes).
      3. Since samples already have loading dye (from GoGreen MM) no need to add loading dye to samples. Place 3-4 uL of each sample into the gel wells using multichannel pipette.
      4. Run gel at 100V with 170mA for 30-40 minutes.
      5. Image using GelRed protocol and on Bio-Rad Gel Doc XR+ imager using Image Lab 5.0.

1. **Clean PCR2 with AMPure XP beads**

**Ampure Preparation:**

- - - 1. Wipe down pipettes and post PCR area with 70% ethanol and/or 10% bleach
      2. Get PCRs from fridge and magnet plate from shelf
      3. Aliquot out Ampure beads to a 1.5 mL tube.
      4. Make fresh 80% ethanol (I find it easiest to open many 1.5 mL tubes, then add 800 ul of 100% ethanol and 200 ul of NF water.)
      5. Make 10mM Tris (pH 8.5) Resuspension Buffer
      6. Gently shake AmPure beads to resuspend (stored in fridge).

**PCR2 Ampure protocol:**

Combine duplicates into one of the PCR tubes.

Add Ampure beads to the tube (**0.7ul beads : 1uL DNA**) and mix by pipetting so solution is homogeneously brown

Let sit 5 minutes at room temperature.

Place on magnet plate. Let sit 2 minutes. Solution should become clear.

Remove ~40 uL of solution so you do not disturb beads.

Add 100 uL of 80% ethanol and let sit 30s.

Remove all ethanol.

Repeat 6 and 7. Remove all ethanol.

Let samples sit 10 minutes in laminar flow hood to allow any residual ethanol to evaporate.

Take tubes off of magnet plate and add 20 uL of Resuspension Buffer (10 mM Tris). Mix by pipetting and let sit 2 minutes.

Let the TRIS sit for a few minutes if the dried beads are hard to re-suspend. Do NOT scrape the sides of the tubes to resuspend dried beads.

Put tubes back on magnet plate and let sit 1 minute.

Transfer elutant to new PCR tube/plate.

***PCR products will degrade over time in the 4C fridge. Move to freezer if not planning to use.

1. **Agilent TapeStation for amplicon sequence length**

Final PCR2 products need to be diluted to 5nM based on the length of the gene region created through PCR. An Agilent TapeStation determines the length of sequences run through the machine.

- - - 1. Use 2-3 samples for this and a ladder on each run.
      2. Use the D1000 ScreenTape System and follow the protocol from the quick guide (<https://www.agilent.com/cs/library/usermanuals/public/ScreenTape_D1000_QG.pdf>)
      3. Base concentrations in the final dilution to 5nM on the base pair length determined from the TapeStation results.

1. **Quantify, Standardize, and Pool PCR2 Products**
   - - 1. Quantify DNA using Qubit fluorometer with 1uL of sample per tube (following Step B protocol).
       2. Dilute PCR2s with Resuspension Buffer (10mM Tris) to 5nM. Conversion of ng/uL to nM uses formula:

(concentration in ng/uL / (660g/mol x average library size in bp) ) x 10^6 = concentration in nM.

***PCR products will degrade over time in the 4C fridge. Move to freezer if not planning to use.

- - - 1. Pool 5uL of 5nM product.
      2. Submit to BNL lab in Elings Hall
      3. All submissions are for amplicon sequencing with average library length 525-539
      4. All sequencing is performed on an Illumina MiSeq with version 2 (v2) chemistry, 500 cycles, paired end
